# The food grade bacterium *Lactobacillus helveticus* VEL12193 promotes autophagy by releasing membrane vesicles

**DOI:** 10.1186/s12964-025-02616-y

**Published:** 2026-01-06

**Authors:** Marie-Agnès Bringer, Simon Manceau, Jana Al Azzaz, Bénédicte Buteau, Lil Proukhnitzky, Amaury Aumeunier, Luis G. Bermúdez-Humarán, Florian Chain, Catherine Daniel, Elise Jacquin, Zandile Mlamla, Jean-Paul Pais de Barros, Julia Novion Ducassou, Yohann Couté, Guilhem Faure, Niyazi Acar, Aurélie Rieu, Pierre Lapaquette

**Affiliations:** 1 Eye & Nutrition Research Group, Centre des Sciences du Goût et de l’Alimentation, Institut Agro, CNRS, INRAE, Université Bourgogne Europe, Dijon, F-21000 France; 2 Université Bourgogne Europe, Institut Agro, INRAE, UMR PAM, Dijon, 21000 France; 3 Laboratory of Commensals and Probiotics-Host Interactions, Université Paris-Saclay, INRAE, Micalis Institute, AgroParisTech, Jouy-en-Josas, 78350 France; 4 Institut Pasteur de Lille, U1019 - UMR 9017 – CIIL Center for Infection and Immunity of Lille, Univ. Lille, CNRS, INSERM, CHU Lille, F-59000 Lille, France; 5 INSERM UMR1231 Center for Translational and Molecular Medicine, Université Bourgogne Europe, Dijon, 21000 France; 6 DiviOmics Platform, UMS 58 BioSand - Université Bourgogne Europe, Dijon, France; 7 LipSTIC Labex, Dijon, France; 8 INSERM, CEA, UA13 BGE, CNRS, CEA, UAR2048, Univ. Grenoble Alpes, Grenoble, 38000 France; 9 Broad Institute of MIT and Harvard, Cambridge, MA 02142 USA; 10 McGovern Institute for Brain Research at MIT, Cambridge, MA 02139 USA

**Keywords:** Autophagy, Membrane vesicles, Probiotic, Ferment, Retina, Gut, Aging

## Abstract

**Background:**

Autophagy-related processes are crucial for maintaining cellular homeostasis in eukaryotic organisms. While alterations of these processes have been strongly linked to specific human disorders, including inflammatory bowel disease, neurodegenerative diseases, and metabolic syndromes, long-term autophagy stimulation appears to be safe and to extend lifespan in model organisms. Several studies indicate that gut microbiota or derived metabolites can modulate host autophagy at the gut mucosa level but also in peripheral organs. Here, we investigated in vitro and in vivo the potential of bacterial species commonly used in food fermentation (ferments) or for their health benefits (probiotics) to modulate host autophagy.

**Methods:**

We screened 11 bacterial strains (lactobacilli and bifidobacteria) in vitro for autophagy induction in human epithelial cells. The most effective strain identified in vitro was then tested in vivo through long-term dietary supplementation in mice to confirm its pro-autophagic effects in the gut and a distant organ, the retina.

**Results:**

In vitro screening of the 11 bacterial strains revealed a strain-dependent ability of bacteria to stimulate autophagy in human epithelial cells. The *Lactobacillus helveticus* strain VEL12193, isolated from cheese, emerged as the strongest autophagy inducer. Long-term dietary supplementation of mice with *L. helveticus* VEL12193 confirmed the pro-autophagic potential of this strain, as evidenced by autophagy stimulation in the gut mucosa but also at distance, in the retina. Finally, we identified membrane vesicles (MVs) from *L. helveticus* as a component involved in bacteria-induced autophagy in epithelial and immune cells, with lactate and specific lipid species potentially contributing to this effect.

**Conclusion:**

In this study, we present evidence that intervention with ferments/probiotics stimulates autophagy in multiple cell types and organs, and we show in vitro that MVs mediate this effect. Additionally, we identify *L. helveticus* VEL12193 as a promising candidate for the development of healthy-aging strategies.

**Supplementary Information:**

The online version contains supplementary material available at 10.1186/s12964-025-02616-y.

## Background

Autophagy is a ubiquitous, highly dynamic eukaryotic cellular process mediating the lysosomal degradation of intracellular cargoes (e.g., organelles, proteins, lipids or intracellular pathogens). The activity level of this process depends on the cell type and varies in response to a wide range of stresses, including metabolic and immune perturbations, in order to maintain cellular homeostasis. At the molecular level, orchestration of the autophagy machinery relies on more than 40 autophagy-related (ATG) proteins with various activities (e.g., kinases, ubiquitin-like conjugation enzymes or lipid transfer proteins). Several types of autophagy can be distinguished. Macroautophagy (hereafter ‘autophagy’) starts with a crescent-shaped phagophore that elongates around its cargo. It then closes to generate a double-membrane autophagosome [1]. Beyond canonical autophagy described above, a growing number of studies describe several non-canonical forms of autophagy involving only a subset of ATG proteins and fulfilling various roles in cells and particularly in cellular membrane dynamic [2, 3].

Autophagy is a keystone of cellular homeostasis by regulating various biological processes such as energy balance, response to oxidative stress, inflammation and cell survival. A decline in autophagy has been repeatedly reported in various organs and model organisms during aging, and dysfunction of this homeostasis gatekeeper is thought to impact multiple features of aging and to contribute to the development of age-related diseases such as age-related macular degeneration (AMD). AMD is a chronic eye disease affecting the macula that is a small retinal area responsible for central vision. AMD is the leading cause of visual impairment and severe vision loss in Western countries, with limited therapeutic options and no curative treatments available. Epidemiological models project that up to 288 million people could be affected worldwide by 2040, although this estimate may vary with demographic and healthcare trends [4–7]. In the retina, autophagy is a crucial process that sustains the function and homeostasis of both the neuroretina and the retinal pigmentary epithelium, which have a high metabolic activity and are particularly exposed to oxidative stress. Alterations of retinal autophagy have been identified in AMD patients and are suspected to be key contributors to the disease [8–11]. Besides age-related diseases, defects in autophagy have also been associated with the onset of other human diseases, including inflammatory bowel diseases (IBDs) such as Crohn’s disease (CD) for which no curative treatment is available [12]. Dysfunction of autophagy in the gut mucosa – a phenotype that can result from polymorphisms and/or mutations in autophagy-related genes (e.g., *ATG16L1*, *IRGM*, *NDP52* or *ULK1*) in patients with CD - results in uncontrolled innate immunity responses including defects in intracellular bacterial killing, decreased antimicrobial peptide secretion, impaired antigen presentation and exacerbated inflammatory response, which are factors contributing to CD [13–16].

The predicted continued rise in the prevalence of IBD and age-related diseases such as AMD makes it crucial to find new levers and to develop innovative preventive strategies. We can assume that strategies focused on supporting autophagy could be beneficial in the context of diseases such as AMD and IBD that involve autophagy defects. Although excessive autophagy can be detrimental in certain contexts (e.g., some cancer cells or autoreactive T cells in lupus may exploit elevated autophagy for survival) [17, 18], chronic and moderate stimulation of autophagy has been shown to increase lifespan and favor healthy aging in various model organisms, suggesting that long-term activation of autophagy is relatively safe and can provide health benefits [19, 20]. However, dietary or therapeutic intervention specifically designed to modulate autophagy for health benefits are not yet available. Autophagy was first described in yeast as a sensor of starvation and other evidence indicates that intermittent fasting or calory restriction in mammals is associated to stimulated autophagy and increased longevity [21]. However, it has been shown that such practices can have harmful effects at the cellular level [22] but also at the organism level (e.g., undernourishment, dehydration) if uncontrolled. Moreover, although several patented pharmacological modulators of autophagy have been described, their precise specificity is unknown and many of them, such as rapamycin analogues or niclosamide, act on multiple intracellular pathways, thus increasing the risk of harmful effects [16, 23]. Thus, developing innovative approaches to stimulate this process remains essential for proposing new strategies to prevent autophagy decline and promote healthy aging.

Interestingly, the gut microbiota can influence autophagy both locally in the gut and remotely in peripheral organs, thus positioning gut microbes as a promising lever to boost autophagy at the scale of the organism [24]. Several microbial-derived products including pathogen-associated molecular patterns (PAMPs; e.g., peptidoglycan, lipoteichoic acids) modulate autophagy in vitro and *in vivo via* their interaction with pathogen recognition receptors (PRRs; e.g., Toll-like receptors, Nod-like receptors [24–26]. Moreover, metabolites produced by microorganisms that act on host metabolism can also influence autophagy, as illustrated in vivo by the inhibitory effect on gut autophagy of the short chain fatty acid (SCFA) butyrate or the stimulatory effect on autophagy of lactate in skeletal muscles [27, 28]. In addition to bacteria, fungi can also exhibit pro-autophagic activities in host cells through cell wall compounds such as β-glucans or *via* the production of metabolites like the disaccharide trehalose [29, 30]. However, although some microbial factors and their associated host cell receptors have been identified, microorganisms displaying pro-autophagic activities still require characterization through functional screening and both in vitro and in vivo validation. Indeed, the regulation of autophagy is cell type-dependent and complex as it involves multiple signaling pathways.

In the present study, we analyzed the potential of 11 food-grade bacterial strains belonging to bacterial species frequently used as probiotics and ferments - lactobacilli and bifidobacteria - to stimulate autophagy. We selected one strain - the *Lactobacillus helveticus* strain VEL12193 - with a promising potential for inducing autophagy and validated its pro-autophagic potential in an in vivo model of aging and in two tissues, the gut mucosa and the retina, the latter serving as proof of concept for an effect occurring at a distance from the gut. Finally, we identified the membrane vesicles (MVs) released by *L. helveticus* VEL12193 as a trigger of autophagy.

## Methods

### Bacterial strains and culture conditions

Bacterial strains used in this study are described in Table 1. Bacteria were grown anaerobically at 37 °C, without shaking, in Man-Rogosa-Sharpe (MRS) medium (Condalab), pH 5.8.

**Table 1:** Bacterial strains used in this study

Organism	Strain number	Origin	Reference
*Lactiplantibacillus pentosus*	VEL12200	Cheese	[31]
*Limosilactobacillus reuteri*	VEL12222	Healthy donor	[31]
*Lactobacillus acidophilus*	VEL12314	Unknown	INRAE collection
*Bifidobacterium infantis*	VEL12148	Infant (intestine)	[32]
*Bifidobacterium longum*	LBH422	Infant (feces)	[33]
*Lacticaseibacillus paracasei*	ATCC334	Cheese	[34]
Lactiplantibacillus plantarum	LBH1062	Pulque	[35]
*Lactobacillus gasseri*	19,992	Healthy donor (feces)	ATCC
*Lactobacillus helveticus*	VEL12193	Cheese	[31]
*Lactiplantibacillus plantarum*	VEL12197	Fermented vegetable	[31]
*Limosilactobacillus fermentum*	NA4	Healthy donor (saliva)	[36]

### Cell lines and treatment with bacteria

Cell lines were maintained at 37 °C in a 5% CO_2_ atmosphere and were routinely tested for mycoplasma contamination (PCR Mycoplasma Test Kit II, PromoKine). The human colonic epithelial cell line HCT116 was obtained from ATCC. The human epithelial HeLa cells stably expressing GFP-LC3 were generously donated by Mathias Faure (Inserm U1111, CIRI, Lyon, France). The authenticity of these cell lines was confirmed by STR profiling (Eurofins). All epithelial cells were maintained in DMEM-GlutaMax (Gibco) supplemented with 10% (vol/vol) fetal bovine serum (FBS, PanBiotech) without antibiotics. The murine macrophage cell line RAW 264.7 was obtained from ATCC and maintained in RPMI medium 1640 (Gibco) supplemented with 10% (vol/vol) FBS (PanBiotech) without antibiotics.

For treatment with bacteria, cells were seeded in 24-well culture plates at a density of 2 × 10^5^ cells per well and incubated for 48 h. Cells were washed twice with phosphate-buffered saline (PBS; pH 7.2), and the medium was replaced by 1 ml of DMEM-GlutaMax supplemented with 10% heat-inactivated FCS. Epithelial cells were treated at a ratio of 10 bacteria per cell for 2–4 h (as indicated in figure legends).

### Antibodies and reagents

Inhibitors of autophagy flux used in the study were bafilomycin A1 (Baf A1, Merck **#**B1793), leupeptin (Merck, **#**L2884**)** and NH_4_Cl (Merck, **#**A9434). L-(+)-Lactic acid (Sigma, #27715–1 L-R) was used to treat the cells. Earle′s Balanced Salts (EBSS) (Sigma, #E2888) was used to stimulate autophagy.

For western blot analyses, the primary antibodies used were anti-LC3B (Merck, **#** L7543), anti-SQSTM1 (p62; Abnova **#**H00008878-M-01), anti-Lamp2a (Abcam, **#**ab18528) and anti-Actin (Merck, **#**A2066). The secondary antibodies used were polyclonal Goat Anti-Rabbit Immunoglobulins/HRP (Agilent, **#**P044801-2) and polyclonal Goat Anti-Mouse Immunoglobulins/HRP (Agilent, **#**P044701-2).

For immunostainings, the primary antibodies used were anti-LC3 (MBL, **#**PM036) and anti-WIPI2 (Millipore, **#**MABC91). Nuclei were stained using DAPI (Merck, **#**D9542).

### Immunofluorescence

For in vitro experiments, cells were seeded on glass coverslips in 24-well plates. Briefly, at the end of the experiment, cells were washed with PBS, fixed with PBS-4% paraformaldehyde for 10 min, and then permeabilized and saturated in PBS-3% BSA-0.05% Triton X-100 for 20 min. Cells were then incubated at room temperature (RT) for 2 h with primary antibodies diluted in PBS-3% BSA, washed and incubated for 1 h at RT with Alexa-fluor conjugated secondary antibodies and DAPI diluted in PBS-3% BSA. Images were acquired using Axiovision Zeiss fluorescent microscope. The number of LC3 or WIPI2 dots per cell was quantified in at least 100 cells per experiment using Icy software [37], with each microscopy image representing a minimum of three independent experiments.

For mouse colon samples, tissues were fixed in 10% buffered formalin, embedded in paraffin, and sectioned. Sections were dewaxed and hydrated by incubating slides successively in xylene, xylene/ethanol, ethanol (100%-50%) and water baths. Antigen retrieval was performed enzymatically by incubating slides in a pre-warmed (37 °C) 0.05% trypsin, 1% NaCl water solution for 15 min. After washing, sections were incubated at RT in Tris Buffered Saline (TBS)-10% FBS-1% BSA for 2 h. Then, sections were incubated overnight at 4 °C with primary antibody, washed twice with TBS-0.025% Tween-20, and incubated with Alexa-fluor conjugated secondary antibody and DAPI in TBS-1% BSA for 1 h at RT. Images were acquired using Axiovision Zeiss fluorescent microscope. The mean intensity of LC3 staining in the gut mucosa was determined using ImageJ software on colon samples from four different mice of each group, with measurements over at least 5 fields for each mouse.

### Autophagy flux assay

#### In vitro experiments

BafA1 was used to inhibit autophagy flux in vitro. BafA1 was added at 100 nM in the cell culture medium during bacteria/cell interaction experiments.

#### Ex vivo experiments

Autophagy flux was assessed ex vivo on dissected retinas and colons. Retinas from the same mouse were placed into two independent wells of a 96-well cell culture plate. One retina was incubated for 4 h in DMEM 4.5 g/L glucose Glutamax^®^ (Pan-Biotech), containing 10% FBS (Biosera) and 10 µg/mL antibiotics (penicillin and streptomycin, Pan-Biotech) while the other retina was incubated for 4 h in the same medium supplemented with 100 µM leupeptin (Merck, L2884) and 20 mM NH_4_Cl (Merck).

For each mouse, the colon was opened longitudinally, and two adjacent areas of the same size were cut out and deposited (intestinal mucosa exposed upwards) into two independent wells on a 96-well cell culture plate. As described for retinas, one of the two pieces of colon was incubated for 4 h in complete cell culture medium without autophagy inhibitors while the other piece was incubated for 4 h in the same medium supplemented with autophagy inhibitors (100 µM leupeptin and 20 mM NH_4_Cl).

At the end of the 4 h incubation period, colon and retina samples were snap-frozen in liquid nitrogen and stored at -80 °C until Western blot analyses.

### Western blot

Whole-cell protein extracts were prepared by directly adding 200 µL of 1.25X Laemmli sample buffer to the cultured cell monolayers, and incubating the lysates for 5 min at 95 °C. Whole-cell protein extracts were prepared from retina and colon samples by using RIPA lysis buffer (Fisher) supplemented with protease and phosphatase inhibitors (PhosSTOP™ and cOmplete™ ULTRA tablets, Merck). Proteins were separated on 4–15% precast polyacrylamide gels (Bio-Rad), transferred on to nitrocellulose membrane (Bio-Rad), blocked for 1 h in TBS containing 5% non-fat dry milk, and probed with primary antibodies overnight at 4 °C and for 2 h with secondary HRP-coupled antibodies at room temperature. Actin level was used to normalize protein quantity. After membrane revelation using ECL detection kit (Perkin Elmer), quantification was performed with ImageJ software.

### RT-qPCR

RNAs were extracted and purified from samples using NucleoSpin RNA/Protein kit (Macherey-Nagel). Reverse transcription was performed with PrimeScript RT reagent kit containing gDNA Eraser (Takara Bio Europe) and using 500 ng of total RNA. Gene expression was analyzed via real-time polymerase chain reaction using SYBR Green (Bio-Rad) on a StepOnePlus™ Real-Time PCR System (Fisher Scientific), with *Hprt* serving as the internal normalization control. Fold induction was calculated with the delta-delta Ct (ΔΔCt) method. Primer sequences are given in Table 2.

**Table 2:** Primers used in this study

Target mRNA (gene ID)	Forward primers	Reverse primers	Ref.
*Atg5* (11793)	GACAGATTTGACCAGTTTTGGGC	GGGTTTCCAGCATTGGCTCTATC	[38]
*Atg7* (74244)	TGCCTATGATGATCTGTGTC	CACCAACTGTTATCTTTGTCC	[39]
*Atg12* (67526)	GGCCTCGGAACAGTTGTTTA	CAGCACCGAAATGTCTCTGA	[40]
*Atg16l1* (77040)	CGAATCTGGACTGTGGATGA	AGCAGGAACTTGGCAGAGAG	This paper
*Becn1* (56208)	GGAAAAGAACCGCAAGGTGGTG	AAACTGTCCGCTGTGCCAGATG	[38]
*Foxo1* (56458)	AGTGGATGGTGAAGAGCGTG	GAAGGGACAGATTGTGGCGA	[41]
*Foxo3* (56484)	GAGGAAAGGGGAAATGGGCA	ACTGTCCACTTGCTGAGAGC	This paper
*Gabarap* (56486)	AATGTCATTCCACCCACCAGT	CGCCACCTCTCTTCGTAGAAT	[42]
*Gabarapl1* (57436)	AGGACCACCCCTTCGAGTAT	AACTGGCCAACAGTGAGGTC	[42]
*Gabarapl2* (93739)	CCGTTGTTGTTGTGGTCGC	GCCCGAGACTTTTTCCACGA	This paper
*Lamp1* (16783)	CAGCACTCTTTGAGGTGAAAAAC	ACGATCTGAGAACCATTCGCA	[43]
*Lamp2a* (16784)	TGGCTAATGGCTCAGCTTTCA	GAACTTCCCAGAGGGGCATC	This paper
*Lc3a* (66734)	TTGGTCAAGATCATCCGGC	GCTCACCATGCTGTGCTGG	[44]
*Lc3b* (67443)	CCCACCAAGATCCCAGTGAT	CCAGGAACTTGGTCTTGTCCA	[45]
*Ndp52* (76815)	TAGTGCTGCTGGCTGCTG	TTGCCATGTTCCAGCAAGGT	This paper
*Optn* (71648)	ATGTCCCATCAACCTCTGAGC	TCAAATCGCCCTTTCATAGCTTG	This paper
*Sqstm1* (18412)	GCTGAAGGAAGCTGCCCTAT	TTGGTCTGTAGGAGCCTGGT	[42]
*Sirt1* (93759)	TATCTATGCTCGCCTTGCGG	TTGTGACACAGAGACGGCTG	[46]
*Sirt2* (64383)	CCACGGCACCTTCTACACAT	TCACACCTGGGAGTTGCTTC	This paper
*Tfeb* (21425)	CCACCCCAGCCATCAACAC	CAGACAGATACTCCCGAACCTT	This paper
*Ulk1* (22241)	AAGTTCGAGTTCTCTCGCAAG	CGATGTTTTCGTGCTTTAGTTCC	[47]
*Uvrag* (78610)	ACATCGCTGCTCGGAACATT	CTCCACGTCGGATTCAAGGAA	[48]
*Vps34* (225326)	CCTGGACATCAACGTGCAG	TGTCTCTTGGTATAGCCCAGAAA	This paper
*Wipi1* (52639)	CTGCTTCTCTTTCAACCAAGACT	ACGTCAGGGATTTCATTGCTT	[49]
*Hprt* (15452)	CAGTCCCAGCGTCGTGATTA	TGGCCTCCCATCTCCTTCAT	[50]

### Mice

All animal experiments adhered to European Union guidelines and the ARVO Statement for the Use of Animals in Ophthalmic and Vision Research, with approval from the French legal and institutional ethics committee review board (2018072513005644). Twelve-month-old male C57BL/6JRj SPF mice were obtained from Janvier Labs, France, and housed at INRAE (C21 231 010 EA) in Dijon, France, under a 12 h:12 h light-dark cycle with ad libitum access to food and water until euthanasia. As previously described [51], mice were randomly assigned to two groups: one receiving a standard diet (*n* = 10) and the other the same diet supplemented with *L. helveticus* (*n* = 10). The dietary regimen was maintained for six months. Before euthanasia, they were fasted for 15 h to induce basal autophagy flux. Euthanasia was performed by cervical dislocation, after which the colon and retina were collected.

### Diet

The *L. helveticus* strain VEL12193 (Table 1) was cultured anaerobically overnight at 37 °C in MRS medium at pH 5.8, without shaking. The bacterial culture was then centrifuged at 5000 g for 10 min at room temperature. The bacterial pellet was washed twice with PBS and resuspended at a concentration of 2 × 10^9^ CFUs/mL in sterile water. As previously reported [51], the bacterial suspension was mixed with SAFE^®^ A04 powdered food for adult mice to reach a bacterial concentration in the diet of 1 × 10^9^ CFU/g. Food portions of 20 g were molded into Petri dishes, dried at 4 °C for 24 h, and stored anaerobically at the same temperature. A fresh batch of the diet was prepared weekly, and food portions were replenished in the cages every two days from the stored stock. The viability of *L. helveticus* in food portions preserved under these conditions was previously verified [51].

### Fractionation of bacterial cell free supernatant (SN) and purification of membrane vesicles

We adapted a protocol from a previous study [52]. A culture of *L. helveticus* VEL12193 (250 mL) was centrifuged at 4000 g and 4 °C for 20 min. The SN was filtered through a 0.45 μm pore size filter (Nalgen Rapid-Flow) to remove remaining bacterial cells and the pH was adjusted to 7.0 with NaOH. Next, the SN was concentrated by ultrafiltration at 4000 g and 4 °C using 100 K Amicon^®^ Ultra-15 centrifugal filters (Merck Millipore). The concentrated SN was filtered through a 0.45-µm-pore-size filter. This fraction constituted the SN *L. helveticus* > 100 kDa (SN *Lh* > 100 kDa). Then, the sample was ultracentrifugated at 125,000 g and 4 °C for 2 h. The resulting pellet of MVs was resuspended in 400 µL of sterile PBS (1X). This fraction constituted the *L. helveticus* MVs (MV *Lh*). The protocol to obtain the fraction SN > 3 kDa of *L. helveticus* (SN *Lh* > 3 kDa) was similar as the one described above except that SN was concentrated by ultrafiltration at 4000 g, 4 °C with 3 K Amicon^®^ Ultra-15 centrifugal filters (Merck Millipore). All fractions were stored as aliquots at − 80 °C for later use. To form the controls (MRS), the same procedures were applied to the MRS culture medium for each fraction. For experiments, fractions were used at a final concentration of 10% (vol/vol) in the cell culture medium to treat epithelial cells (GFP-LC3 Hela cells) or macrophages (RAW 264.7).

### Transmission electron microscopy

Five microliters of sample (MV *Lh* or MRS) were applied and incubated for 1 min on carbon-coated Formvar 200 glow discharge grids. Grids were blotted and then stained with Uless solution ^®^ (Delta microscopies, USA) for 2 min. Visualizations were performed on the DimaCell platform (http://www.dimacell.fr/) using a transmission electron microscope (HITACHI HT7800) operating at 80 kV and equipped with two AMT cameras (NanoSprint43 AMT, Woburn, USA).

### Dynamic light scattering (DLS) measurement

Dynamic light scattering (DLS) measurements were carried out using Submicron Particle Sizer NICOMP 380 on the DIVVA platform (https://umr-pam.fr/plateformes-technologiques/plateforme-divva/pole-aliments). MVs were loaded into disposable cells, and data collected at 25 °C. All samples were prepared in 1X PBS buffer, pH 7.4, and filtered at 0.22 μm before use. For each sample, the autocorrelation function was an average of three runs of 10 min each, repeated three times.

### Mass spectrometry-based proteomic analyses

Three independent extracellular vesicles preparations were analyzed. Proteins solubilized in Laemmli buffer were stacked in the top a NuPAGE 4–12% gel (Invitrogen) before Coomassie brilliant blue R-250 staining (Biorad). Proteins were then digested in-gel using modified trypsin (sequencing purity, Promega) as previously described [53], except that Tris(2-carboxyethyl)phosphine hydrochloride was used instead of dithiothreitol. The resulting peptides were analyzed by online nanoliquid chromatography coupled to MS/MS (Ultimate 3000 RSLCnano and Orbitrap Exploris 480, Thermo Fisher Scientific). For this purpose, the peptides were sampled on a precolumn (300 μm x 5 mm PepMap C18, Thermo Scientific) and separated in a 75 μm x 250 mm C18 column (Aurora Generation 3, 1.7 μm, IonOpticks) using a 60 min acetonitrile gradient. The MS and MS/MS data were acquired by Xcalibur version (version 4.2, Thermo Fisher Scientific).

Peptides and proteins were identified by Mascot (version 2.8.3, Matrix Science) through concomitant searches against the Uniprot database (*Lactobacillus helveticus* taxonomy, 20241115 download) and a homemade database containing the sequences of classical contaminant proteins found in proteomic analyses (keratins, trypsin, etc.). Trypsin/P was chosen as the enzyme and two missed cleavages were allowed. Precursor and fragment mass error tolerances were set at respectively at 10 and 20 ppm. Peptide modifications allowed during the search were: Carbamidomethyl (C, fixed), Acetyl (Protein N-term, variable) and Oxidation (M, variable). The Proline software (version 2.3.1 [54]), was used for the compilation, grouping and filtering of the results (conservation of rank 1 peptides, peptide length ≥ 6 amino acids, false discovery rate of peptide-spectrum-match identifications < 1% [55], and minimum of one specific peptide per identified protein group). Proline was then used to perform a MS1-based label-free quantification of the identified protein groups. For each protein groups, intensity-based absolute quantification (iBAQ [56]), values were calculated from MS1 intensities of specific and razor peptides.

For bioinformatic analyses, the total list of identified proteins was filtered based on the following criteria: (i) sequence coverage ≥ 15%, (ii) detection of at least two unique peptides, (iii) quantification in at least two out of three biological replicates, (iv) coefficient of variation (CV) ≤ 60% across replicates, (v) fold change ≤ 5 between replicates, and (vi) abundance within the top 80% (≥ 20th percentile) of detected proteins. This process yielded a final high-confidence set of 432 proteins. Comprehensive protein domain and feature analyses were performed using InterProScan 5 [57], which integrates multiple predictive algorithms and databases. Signal peptide predictions were conducted using Phobius [58], and transmembrane (TM) segment predictions were performed using TMHMM [59]. Functional classification of the filtered proteins was performed using the Clusters of Orthologous Groups (COG) database [60]. Protein sequences were analyzed via RPS-BLAST [61, 62] against the COG-24 release on the NCBI Conserved Domain Database platform [63]. Only specific hits with an E-value cutoff of 0.01 were retained to ensure reliable functional assignments, excluding non-specific matches. COG identifiers were mapped to functional categories, with category letters linked to full functional descriptions. This approach annotated 354 proteins (81.9% of the filtered dataset), corresponding to 356 functional assignments distributed across 20 COG categories.

### Quantitative targeted LC-MS lipidomics

*L. helveticus* MVs (quantity based on ~ 200 µg of protein) and MRS-derived MVs (quantity based on ~ 100 µg of protein) were spiked with 2.5 µL of the Ultimate^®^ONE SPLASH standard mix (Ref 330820 L), and 10µL of an internal standard mix containing tetramyristoyl cardiolipin (200 ng), monogalactosyldioleoylglycerol (d18) (500 ng), and digalactosyldioleoylglycerol (d18) (750 ng). The SPLASH mixture contained 69 authentic deuterated internal standards with concentrations ranging from 25 to 150 ng/µL. Total lipids were extracted with the acidified Bligh & Dyer method [64].

Lipid extracts (4 µL) were loaded onto a Zorbax^®^Eclipse Plus C18 1,8 μm, 2,1 × 100 mm column maintained at 55 °C (Agilent Technologies) for all complex lipids with the exception of cardiolipins (CL). For the analyses of CL, lipid extracts (4 µL) were loaded onto a Phenomenex Luna [2] C18 3 μm, 2 × 250 mm column which also was maintained at 55 °C. Phosphatidylglycerols (PG), digalactosyldiacylglycerols (DGDG), monogalactosyldiacylglycerols (MGDG), diacylglycerols (DG), phosphatidylethanolamines (PE), phosphatidylcholines (PC), phosphatidylinositol (PI), and phosphatidylserines (PS) were analyzed by LC-MS/MS. For the analysis of the aforementioned lipid families except for CL, PC, and PE, the Agilent 1260 Infinity LC system coupled to an Agilent 6460 Triple Quadrupole LC-MS, equipped with an electrospray ionization source (Agilent Technologies) was used. Mobile phase A (acetonitrile/water/1 M ammonium formate (60/39/1 v/v/v) with 0.1% formic acid), and mobile phase B (isopropanol/acetonitrile/1 M ammonium formate (90/9/1 v/v/v) with 0.1% formic acid) were used at a flow rate of 0.4 mL/min for the elution of DGDG, MGDG, DG, PG, PI and PS [65]. For the separation of DGDG, MGDG and DG, the gradient was as follows: 2 min hold at 0% B; 0–20% B in 4 min; 2 min hold at 20%; ramp-up to 40% in 0.1 min; 40–65% B in 11.9 min; ramp-up to 75% in 0.1 min; 75–99% B in 1.9 min; 2 min hold at 99%, 99%-0% ramp-down in 0.1 min; return to initial conditions in 3.9 min. A flow rate of 0.25 mL/min was used for the separation of PG, PI and PS. For the separation of PG, the gradient was as follows: 2 min hold at 0% B; 0–20% B in 2 min; 4 min hold at 20%; ramp-up to 40% in 0.1 min; 40–65% B in 11.9 min; ramp-up to 75% in 0.1 min; 75–99% B in 1.9 min; 2 min hold at 99%, 99%-0% ramp-down in 0.1 min; return to initial conditions in 3.9 min. For the separation of PI and PS, the gradient was as follows: 1 min hold at 40% B; 40–95% B in 14 min; 1 min hold at 99%, 99%-40% ramp-down in 1 min; return to initial conditions in 3 min. TG separation was achieved with mobile phase A (acetonitrile/water/1 M ammonium acetate (60/39/1 v/v/v) with 0.1% formic acid), and mobile phase B (isopropanol/acetonitrile/1 M ammonium acetate (90/9/1 v/v/v) with 0.1% formic acid) [65] at a flow rate of 0.4 mL/min. The gradient was set as follows: 1 min hold at 30% B; 30–60% B in 2 min; 60–72% B in 15 min; 72–99% B in 20 min; 3 min hold at 99%, 99%-30% ramp-down in 0.1 min and maintained at 30% B for 3.9 min. With the exception of TG, the acquisition source parameters were as follows: nebulizer gas flow rate 20 L/min, sheath gas 11 L/min, capillary 3500 V, nozzle 1000 V, sheath gas temperature 220 °C and set at 300 °C for PI and PS). For TG, the source parameters were as follows; nebulizer gas flow rate 15 L/min, sheath gas 11 L/min, sheath gas temperature 150 °C, capillary 4500 V, nozzle 1200 V. The source temperature was set at 250 °C for DGDG, MGDG and DG, 200 °C for PG, 150 °C for TG, and at 325 °C for PI and PS. The fragmentor was set at 148 V for DGDG and DG, 160 V for MGDG, 185 V for PG, 220 V for TG, 172 V for PI and 150 V for PS. The collision energy was at 23 V for DGDG and DG, 22 V for MGDG, 42 V for PG, 25 V for TG, 50 V for PI and 19 V for PS. NH4^+^-DGDG and NH4^+^-MGDG were quantified by monitoring the product ion which results from the neutral loss of 359 Da [2 Gal + H2O + NH3] and 197 Da [1 Gal + H2O + NH3] [66]. Monoglyceride ions of DGDG and MGDG were used for structural and retention time confirmation. NH4^+^-DG were detected and quantified based on two product ions (monoglyceride ions), resulting from the neutral loss of either the *sn*-1 (*sn*-1 FA + H2O + NH3) or *sn*-2 (*sn*-2 FA + H2O + NH3) fatty acids. The same was the case for the detection of NH4^+^-TG, except loss of the *sn*-3 (*sn*-2 FA + H2O + NH3) fatty acid was also considered. NH4^+^-DG and NH4^+^-TG were quantified according to the sum of the responses. H–PG were quantified according to the sum of responses resulting from their fatty acid fragment ion(s). H–PS and H–PI were respectively quantified based on the neutral loss of the 87 Da serine residue and the 241 Da phosphoinositol fragment ion. Lipid concentrations were then determined by calculating the relative responses of each respective lipoform to a corresponding internal standard selected based on its carbon chain length and unsaturation status. For the analyses of PC, PE and CL, the Vanquish LC system, coupled to a triple-stage quadrupole (TSQ) Altis mass spectrometer, with a heated electrospray ionization source (Thermo Scientific) was used. For the analyses of PC and PE, Mobile phase A (water/methanol (60/40 v/v) modified to a pH of 5 with 10mM ammonium acetate and acetic acid), and mobile phase B (isopropanol/methanol (90/10 v/v) modified to a pH of 6.6 with 10mM ammonium acetate and acetic acid) were used. For PC and PE, a gradient at a flow rate of 0.2 ml/min was as follows: 1 min hold at 35% B; 35–95% B in 14 min; 1 min hold at 95%, 95%-35% ramp-down in 1 min; return to initial conditions in 3 min. For the separation of CL, mobile phase A (acetonitrile/water (90/10 v/v)), and mobile phase B (isopropanol/methanol (90/10 v/v)), with both mobile phases buffered with 0.5% acetic acid and 0.2% ammonia were used [67]. A gradient at a flow rate of 0.25 ml/min was as follows: 5 min hold at 50% B; 50–90% B in 10 min; 90%-50% ramp-down in 0.1 min; return to initial conditions in 6.9 min. The ion source parameters for the H-ESI probe were set as follows: sheath gas, 50 arb; auxiliary gas, 10 arb; sweep gas, 1 arb; ion transfer tube temperature, 325 °C; vaporizer temperature, 350 °C and ion spray voltage, 3500 V (+), and 2500 V (-). Data analyses was achieved with the TraceFinder 4.1 General Quantitative software (Thermo Scientific). H–CL molecular species were quantified based on their fatty acid fragment ion(s). With the exception of tetra-acyl variants, detection of a minimum of two fatty acid fragment ions for cardiolipin molecular species was required. Lipid concentrations were determined by calculating the summed response ratio(s) of the contributing fatty acid(s) with regards to (14:0)_4_CL. PC were quantified based on the 184.1 phosphocholine product ion, whereas PE were detected based on the neutral loss of their 141 Da phosphoethanolamine ion [68]. Lipid concentrations were determined by calculating response ratios with regards to deuterated PC or PE internal standards.

Lipid concentrations for all quantified lipid classes were expressed in pmol/µg of Protein.

### Staining of MVs to monitor their uptake and trafficking in host cells

MVs were purified as described above with the exception of the pellet, which was resuspended, diluted ten-fold, and stained with 1% Vybrant DiI cell-labeling (Invitrogen, V22889) at 37 °C for 30 min. The solution was then added on the top of a gradient (OptiPrep™, D1556) and Tris-sucrose buffer (0.25 M sucrose, 10.02 µM Tris-HCl, pH 7.0) and ultracentrifugated at 100,000 g and 4 °C for 18 h. The stained MVs were collected between the layers 20%-40% of iodixanol, resuspended in PBS and ultracentrifugated at 125,000 g and 4 °C for 2 h. Finally, the pellet was resuspended in 200 µL of PBS. As a control, the same procedure was applied to the purification product carried out on growth medium (MRS). Purified stained MVs were added at a final concentration of 5% (vol/vol) in the cell culture medium of HeLa-GFP LC3 cells. A centrifugation step (200 g,10 min) enabled to facilitate the contact of the EVs with the cells. The cells were observed by fluorescent microscopy after a 6 h incubation with MVs at 37 °C.

### Statistical analyses

Statistical analyses were performed using Prism 9 software (GraphPad Software Inc.). ANOVA one way, Wilcoxon, Kruskal-Wallis and Mann-Whitney tests were used in this study. All *p*-values of below 0.05 were considered statistically significant.

## Results

### In vitro screening of the ability of lactobacilli and bifidobacteria strains to stimulate autophagy

**Fig. 1:**
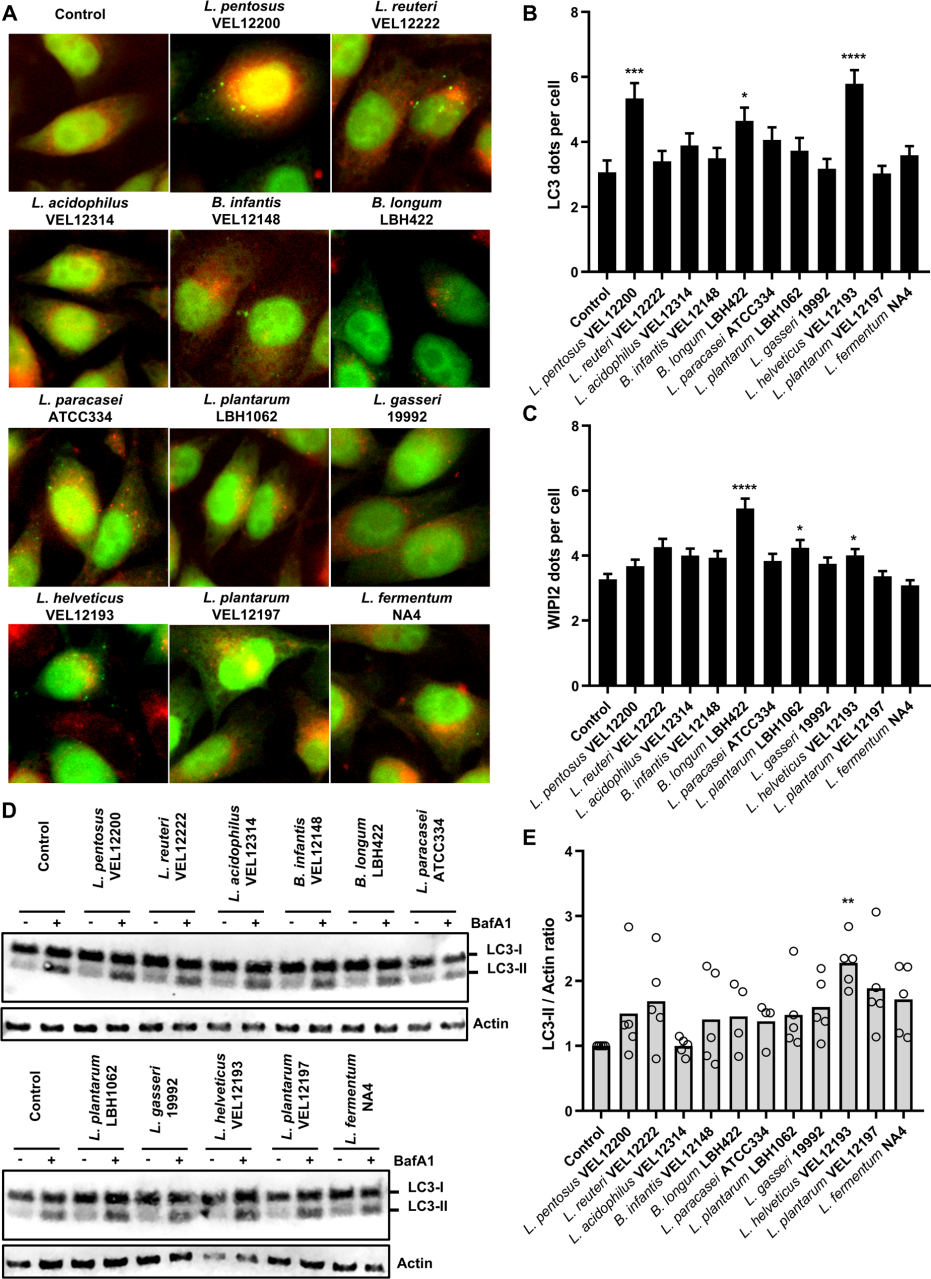
Strain-dependent stimulation of autophagy in Hela GFP-LC3 cells. **A** Representative images of GFP-LC3 HeLa cells treated or not (control) for 2 h with 11 different strains of lactobacilli and bifidobacteria. Cells were immunostained with anti-WIPI2 antibody (red). The GFP-coupled LC3 protein (GFP-LC3) appears in green. **B** and **C** Quantification of the number of (**B**) LC3 and (**C**) WIPI2 dots per cell 2 h after treatment of GFP-LC3 Hela cells with bacteria. Data are mean ± SEM of three independent experiments. **D** and **E** In vitro autophagy flux assay. **D** Representative immunoblots of GFP-LC3 HeLa cells treated or not (control) with 11 different strains of lactobacilli and bifidobacteria for 2 h, in presence or absence of the autophagy flux inhibitor bafilomycin A1 (BafA1). Immunoblot analyses were performed using anti-LC3B and anti-actin antibodies. **E** The LC3-II/Actin ratio was calculated and normalized to that obtained in control cells without BafA1. Data are mean ± SEM of five independent experiments. **B**, **C** and **E** Kruskal-Wallis test (* *p* < 0.05, ** *p* < 0.01, *** *p* < 0.001 and **** *p* < 0.0001)

Eleven lactobacilli and bifidobacteria, which are Gram-positive lactic bacteria, were analyzed for their ability to modulate autophagy in vitro in human epithelial cells (Table 1). To increase the robustness of this phenotypic analysis, we combined several approaches. First, we compared the level of autophagy activation induced by the different bacterial strains by counting the number of intracellular vacuoles (appearing as dots on images) positive for two autophagy markers - LC3 and WIPI2 - in HeLa GFP-LC3 reporter cells by imaging. LC3 proteins are present on autophagic vacuoles from initial to late stages of the process, while WIPI2 proteins are only present on the isolation membrane, at initial stages [69]. We observed that two strains, namely *Bifidobacterium longum* LBH422 and *L. helveticus* VEL12193, were the only ones among the 11 tested to induce both a significant increase in the number of LC3 and WIPI2 dots compared to untreated cells (Fig. 1A-C). As illustrated in *L. helveticus* VEL12193 strain-treated cells, the two markers sometimes colocalized indicating the production of nascent autophagosomes (Sup Fig. 1). Notably, none of the strains tested decreased the number of LC3 or WIPI2 dots per cell compared to untreated cells, suggesting that these bacteria have no inhibitory effects on autophagy, at least on the initial steps of the process. 

The marked increase in LC3 dots associated with *B. longum* LBH422 and *L. helveticus* VEL12193 cell treatment could be due to either induction of the autophagy process (i.e., formation of new autophagosomes) or blockade in the latter stages of the process (inhibition of autophagosomes maturation), both leading to accumulation of autophagic vacuoles and hence lipidation of LC3. Therefore, to avoid misinterpretation, we analyzed the autophagic flux by using BafA1 - an autophagy flux inhibitor that prevents autophagosome-lysosome fusion - and we assessed the accumulation rate of endogenous LC3-II, the lipidated form of LC3 that is recruited on autophagic vacuole [70]. Accumulation of LC3-II in cells treated with bacterial strains compared to untreated cells would suggest a stimulation of the autophagy process. We observed that *L. helveticus* VEL12193 was the only one to induce a significant accumulation of LC3-II in HeLa cells compared to untreated cells (Fig. 1D-E). Since *B. longum* LBH422 treatment failed to increase autophagic flux, this strain was not retained for further characterization of its autophagy-modulatory properties.

Taken together, these results showed that not all strains of lactobacilli and bifidobacteria could stimulate autophagy in epithelial cells in vitro. Among the 11 tested strains, *L. helveticus* strain VEL12193 was identified as a promising autophagy inducer.

**Fig. 2:**
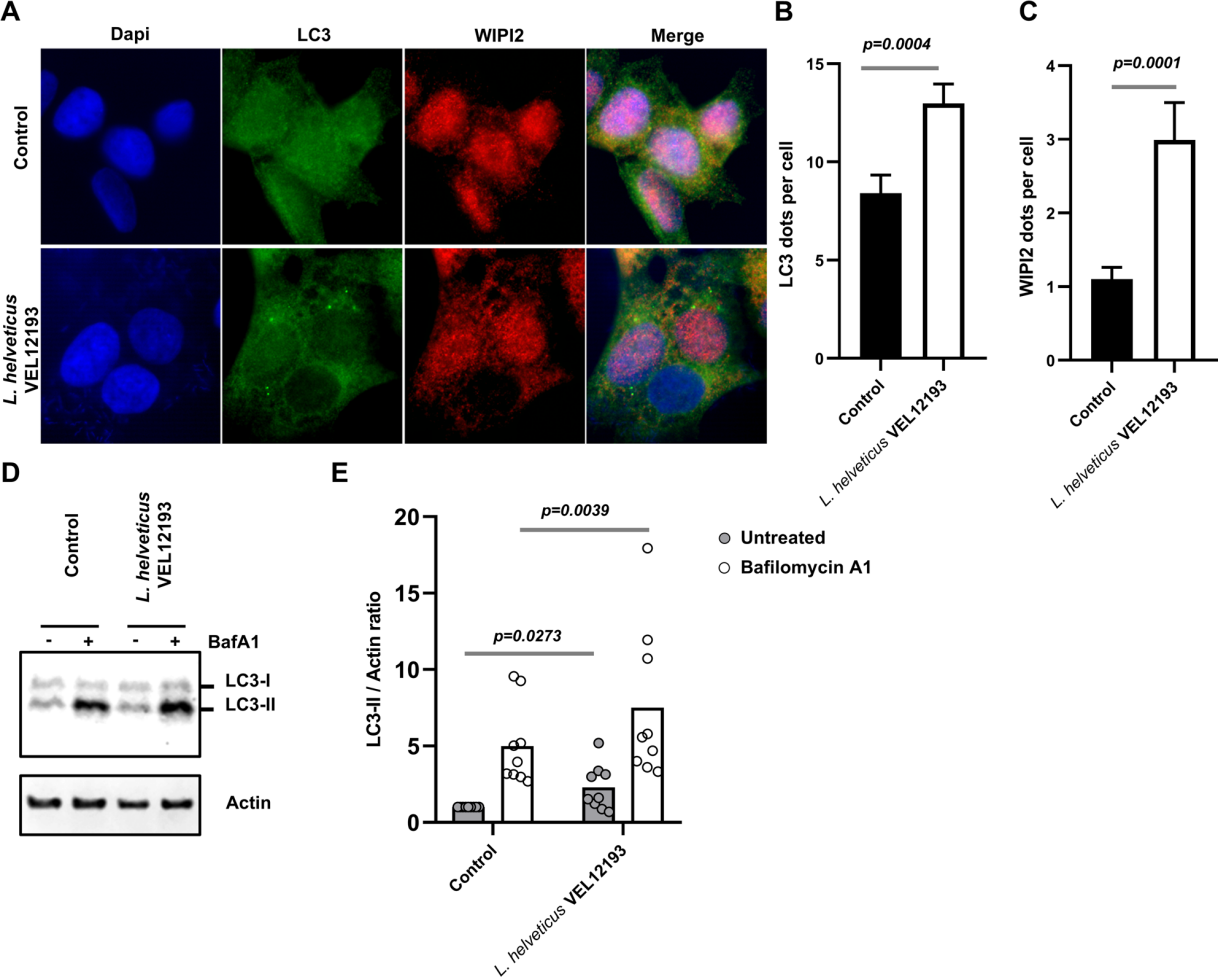
*L. helveticus* strain VEL12193 stimulates autophagy in HCT116 human intestinal epithelial cells. **A** Representative images of HCT116 cells treated or not (control) for 2 h with the *L. helveticus* strain VEL12193. Cells were immunostained with anti-LC3 (green) and anti-WIPI2 (red) antibodies. Nuclei were stained with DAPI (blue). **B** and **C** Quantification of the number of (**B**) LC3 and (**C**) WIPI2 dots per cell 2 h after treatment of HCT116 cells with bacteria. Data are mean ± SEM of three independent experiments. Mann-Whitney test was used and *p*-values are indicated on the graphs. **D** and **E** In vitro autophagy flux assay. **D** Representative immunoblots of HCT116 treated or not (control) with *L. helveticus* strain VEL12193 for 2 h, in presence or absence of bafilomycin A1 (BafA1). Immunoblot analyses were performed using anti-LC3B and anti-actin antibodies. **E** The LC3-II/Actin ratio was calculated and normalized to that obtained in control cells without BafA1 Data are mean ± SEM of nine independent experiments. Wilcoxon matched pairs signed rank test was used and *p*-values are indicated on the graphs

To validate the pro-autophagy property of *L. helveticus* VEL12193, we repeated similar experiments in the human intestinal epithelial cell line HCT116, which are commonly used to study autophagy [3, 71, 72]. A significant increase in the number of intracellular vacuoles positive for LC3 and WIPI2 was observed in HCT116 cells treated with *L. helveticus* VEL12193 compared to untreated cells (Fig. 2A-C). In addition, cell treatment with *L. helveticus* VEL12193 induced a significant increase in LC3-II accumulation compared to untreated cells (Fig. 2D-E). Thus, these results confirmed the pro-autophagy property of *L. helveticus* VEL12193 in vitro.

### Effect of a long-term dietary supplementation of *L. helveticus* VEL12193 on intestinal autophagy in mice

Next, we explored whether *L. helveticus* strain VEL12193 can enhance autophagy in vivo in mice. Since activity of autophagy declines with aging [4], we evaluated the ability of a long-term supplementation (6 months) to stimulate autophagy in aged mice (18 months of age at the end of the experiment). For such a long-term protocol, it was not conceivable ethically to administer the bacteria by oral gavage. Thus, we chose to incorporate the bacteria in the food by formulating pellets enriched with *L. helveticus* VEL12193. We previously showed that supplementation of *L. helveticus* VEL12193 strain was well-tolerated by mice with no noticeable side effect and was associated with an enrichment of mouse feces with *L. helveticus* DNA [51].

**Fig. 3:**
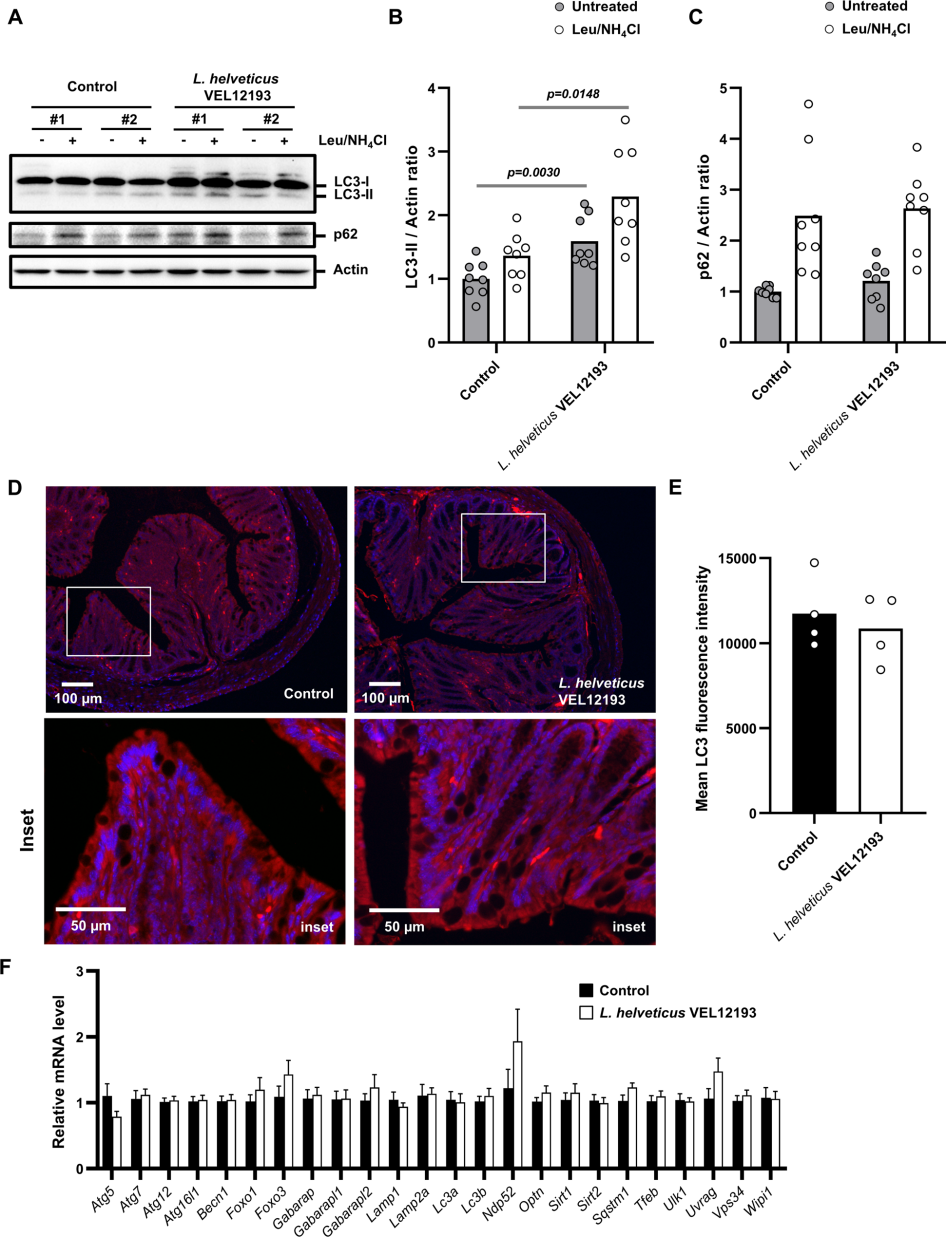
Long-term dietary supplementation of mice with *L. helveticus* VEL12193 prevents autophagy decline in the gut mucosa during aging. **A**-**C** Ex vivo autophagy flux assay. **A** Representative immunoblots of colonexplants from mice fed a control or *L. helveticus* VEL12193-supplemented diet. Explants from same mouse were incubated or not ex vivo with autophagy inhibitors (leupeptin (Leu) and NH4Cl). Immunoblot analyses were performed using anti-LC3B, anti-p62 and anti-actin antibodies. **B** and **C** The LC3-II/Actin (**B**) and p62/Actin (**C**) ratio were calculated and normalized to those obtained in control colonic explants untreated with autophagy flux inhibitors. Data are mean ± SEM (*n* = 8 mice/group). Mann-Whitney test was used and p-values are indicated on the graphs. **D** Representative images of colon sections from mice fed a control or *L. helveticus* VEL12193-supplemented diet. White squares in upper panels indicate inset areas displayed in the corresponding lower panels. Samples were immunostained with anti-LC3 antibodies (red) and nuclei were stained with Hoescht (blue). **E** The mean intensity of LC3 staining in colon mucosa sections was determined using ImageJ software. *n* = 4 mice/group (five fields per mouse section were analyzed). Mann-Whitney test was used. **F** Expression of genes encoding proteins involved in the autophagy pathway in the colon of mice fed a control diet (*n* = 9) or fed a diet supplemented with *L. helveticus* VEL12193. mRNA levels were normalized to Hprt mRNA to determine relative transcript levels. Data are mean +/- SEM (*n* = 8 mice/group). Mann-Whitney test was used

Autophagy is a highly dynamic process whose activity remains difficult to assess in vivo [73]. As the intestinal compartment is the one through which bacteria-supplemented food transits, we first studied autophagy in the mouse gut mucosa. Analysis of the autophagy flux in colon explants revealed that long-term consumption of food enriched with *L. helveticus* VEL12193 significantly increased the level of LC3-II in the mouse colon at both, the basal level (without treatment with the autophagic flux inhibitors leupeptine (Leu) and NH_4_Cl) and when the autophagy flux was blocked by inhibitors (Leu/ NH_4_Cl) compared to control mice (Fig. 3A-B). The trend in p62 protein accumulation – an autophagy receptor degraded in mature autophagosomes - in explants treated with autophagy flux inhibitors compared to untreated explant confirmed the efficacy of the autophagy flux blockade by the drugs (Fig. 3A and C). However, we did not observe any significant difference in the accumulation of this autophagy marker according to the dietary supplementation of mice with *L. helveticus* VEL12193 (Fig. 3A and C).

It is technically difficult to assess subtle changes in the number of autophagic vacuoles by immunofluorescence on tissue sections. Immunostaining of colon sections with LC3 antibodies revealed a heterogeneous expression of this marker in the gut mucosa characterized by the presence of cells highly positive for this autophagy marker. Analysis of mean LC3 fluorescence intensity showed no significant difference between colon sections from mice supplemented with *L. helveticus* VEL12193 and those from untreated mice (Fig. 3D-E).

Since autophagy is regulated at the transcriptional level [74], we compared the expression level of 24 genes encoding autophagy-related proteins (ATGs) in the colonic mucosa of mice supplemented with *L. helveticus* and control mice (Fig. 3F). Six-month dietary supplementation with *L. helveticus* was not associated with modulation of the expression of ATG genes including genes encoding proteins involved in the transcriptional control of autophagy (*Foxo1*, *Foxo3*, *Sirtuin 1*, *Sirtuin 2* and *Tfeb*), in initiation steps (*Becn1*, *Ulk1*, *Uvrag*, *Vps34* and *Wipi1*), in selective forms of autophagy (*Ndp52*, *Optineurin* and *p62*), in conjugation systems required for elongation of the phagophore (*Atg5*, *Atg7*, *Atg12*, *Atg16l1*, *Gabarap*, *GabarapL1*, *GabarapL2*, *Lc3a* and *Lc3b*) and in maturation (*Lamp1* and *Lamp2a*).

Although we did not observe any activation of autophagy at the transcriptional level, our results revealed that long-term consumption of *L. helveticus* VEL12193 stimulated autophagy flux in the colon of 18-month-old mice. This suggests that consumption of this bacterial strain could promote autophagy activity in the gut mucosa of old mice.

### Impact of long-term consumption of *L. helveticus* VEL12193 on autophagy at the extraintestinal level: the retina as a study model and proof of concept

**Fig. 4:**
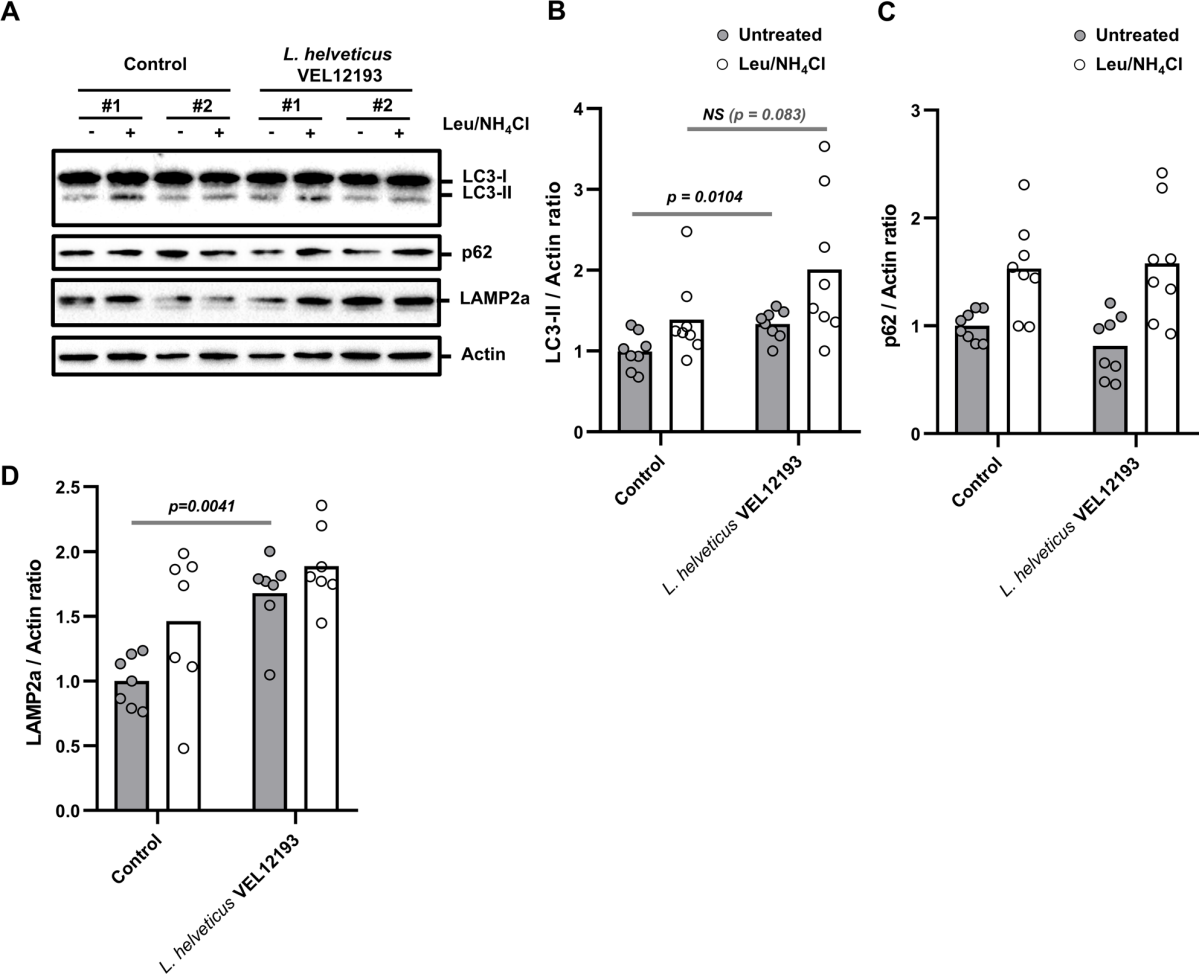
*L. helveticus* VEL12193 stimulates autophagy in the retina. **A**-**D** Ex vivo autophagy flux assay. **A** Representative immunoblots of retinal explants from mice fed a control or *L. helveticus* VEL12193-supplemented diet incubated or not ex vivo with autophagy inhibitors (leupeptin (Leu) and NH4Cl) for 4 h. Immunoblot analyses were performed using anti-LC3B, anti-p62, anti-LAMP2a and anti-actin antibodies. **B**, **C** and **D** The LC3-II/Actin (**B**) p62/Actin (**C**) and LAMP2a/Actin (**D**) ratio were calculated and normalized to levels obtained in retinal explants untreated with autophagy flux inhibitors (*n* = 8 mice/group). Mann-Whitney test was used and *p*-values are indicated on the graphs

Evidence has accumulated in recent decades showing that gut microbiota influences autophagy in peripheral organs [24, 75]. Thus, we investigated whether long-term dietary supplementation with *L. helveticus* VEL12193 modulates autophagy in the retina of aged mice (Fig. 4). Autophagy flux was analyzed ex vivo in the retinas of 18-month-old mice with or without *L. helveticus* supplementation over a 6-month period. A significant increase in the basal level of LC3-II, along with a near-significant trend of LC3-II accumulation under condition where the autophagy flux is blocked by autophagy inhibitors (Leu/ NH_4_Cl), were observed in retinas of *L. helveticus*-supplemented mice compared with those of control animals (Fig. 4A-B). Similar to our observations in the colonic mucosa, treating retinas with autophagy inhibitors resulted in an increase in p62 level compared to untreated cells indicating that the autophagy flux was blocked by the drugs. However, in this tissue, again, long-term consumption of *L. helveticus* VEL12193 had no effect on p62 accumulation (Fig. 4A and C).

Chaperone-mediated autophagy (CMA) is a form of autophagy that ensures the selective degradation of cellular proteins tagged with a KFERQ-like motif by lysosomes [76]. Crosstalk regulation exists between macroautophagy (generically referred to as autophagy) and CMA [77]. It has been reported that the reduction in macroautophagy activity observed during aging in the retina coincides with an increase in CMA. This cross talk is unidirectional in retinal cells and is thought to preserve retinal homeostasis. The lysosomal protein LAMP2a is commonly used as a marker of CMA [78, 79]. Interestingly, the basal level of LAMP2a was significantly higher in retinas of *L. helveticus*-supplemented mice, compared to those of control mice (Fig. 4A and D).

Taken together, these results suggest that long-term consumption of *L. helveticus* VEL12193 favors both, macroautophagy and CMA in the retina of old mice.

### Involvement of membrane vesicles released by *L. helveticus* VEL12193 in autophagy stimulation

Health benefits associated with lactic acid bacteria are mediated, at least in part, by compounds or metabolites produced and secreted by these bacteria [80]. To gain insight into the molecular mechanisms by which *L. helveticus* VEL12193 stimulates autophagy, we investigated the ability of *L. helveticus* VEL12193 culture supernatant (SN) to trigger such an effect.

**Fig. 5:**
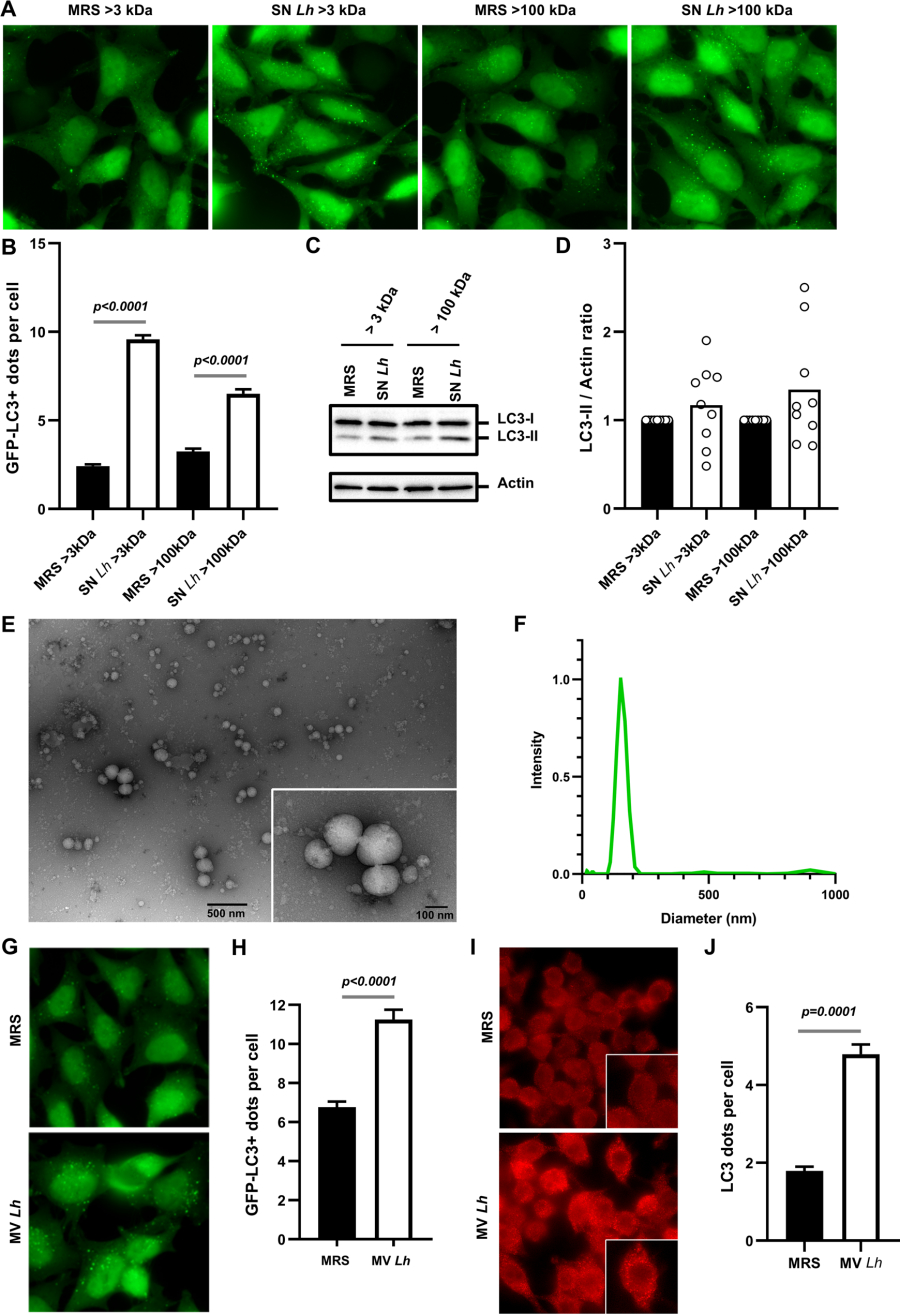
Membrane vesicles released by *L. helveticus* VEL12193 stimulate autophagy in epithelial cells. **A** Representative images of GFP-LC3 HeLa cells treated for 2 h with fractions (> 3 kDa or > 100 kDa) of *L. helveticus* supernatant (SN Lh) or MRS medium (bacterial culture medium, control). The GFP-coupled LC3 protein (GFP-LC3) appears in green. **B** Quantification of the number of LC3 dots per cell at 2 h post treatment in GFP-LC3 Hela cells. Data are mean ± SEM of three independent experiments. Mann-Whitney test was used and *p*-values are indicated on the graph. **C** In vitro autophagy flux assay. Representative immunoblots of GFP-LC3 HeLa cells treated or not (control) with the fractions (> 3 kDa or > 100 kDa) of *L. helveticus* SN (SN Lh) for 2 h, in presence of the autophagy inhibitor bafilomycin A1 (BafA1). Immunoblot analyses were performed using anti-LC3B and anti-actin antibodies. **D** The LC3-II/Actin ratio was calculated and normalized to that obtained in control cells without BafA1. Data are mean ± SEM of nine independent experiments. Wilcoxon matched pairs signed rank test was used. **E** Negative-staining transmission electron microscopy image of MVs purified from the SN of *L. helveticus* VEL12193. **F** Size measurement of unlabeled MVs from *L. helveticus* VEL12193 by dynamic light scattering. **G** Representative images of GFP-LC3 HeLa cells treated for 2 h with MVs purified from *L. helveticus* SN or MRS medium (control). The GFP-coupled LC3 protein appears in green. **H** Quantification of the number of LC3 dots per cell 2 h after treatment of GFP-LC3 Hela cells with MVs. Data are mean ± SEM of three independent experiments. Mann-Whitney test was used and *p*-value is indicated on the graph. **I** Representative images of RAW264.7 macrophages treated for 2 h with MVs purified from the SN of *L. helveticus* (MV Lh) or bacterial culture medium (MRS medium, control). Cells were immunostained with anti-LC3 antibody (red). **J** Quantification of the number of LC3 dots per cell 2 h after treatment of RAW264.7 macrophages with MVs. Data are mean ± SEM of three independent experiments. Mann-Whitney test was used and the *p*-value is indicated on the graph

For this purpose, the cell-free supernatant of *L. helveticus* (SN *Lh*) and its growth medium (MRS, control) were concentrated by size-exclusion ultrafiltration with a 3 kDa (> 3 kDa) or a 100 kDa (> 100 kDa) molecular weight cutoff. Treatment of GFP-LC3 Hela cells with the SN *Lh* > 3 kDa fraction was associated with a significant increase in the number of GFP-LC3 positive dots (Fig. 5A-B) and trend toward an increase in the accumulation of LC3-II in BafA1-treated cells compared to cells treated with the control fraction (MRS > 3 kDa ) (Fig. 5C-D). Interestingly, these phenotypes were still observed with the SN *Lh* > 100 kDa fraction (Fig. 5A-D), suggesting that a bacterial compound with a high molecular weight is involved in the ability of *L. helveticus* VEL12493 to stimulate autophagy.

We hypothesized that MVs, which are lipid nanoparticles of high molecular weight able to interact with host cells and to modulate their activities, might be involved in the stimulation of autophagy by *L. helveticus* [81, 82]. To our knowledge, there are no reports demonstrating the release of MVs by the bacterial species *L. helveticus*. Therefore, we first investigated whether *L. helveticus* strain VEL12193 produces such nanoparticles. Analysis of concentrated and purified *L. helveticus* SN revealed the presence of spherical structures with an average diameter of about 150 nm, with most vesicles between 120 nm and 200 nm in diameter (Fig. 5E-F). Such structures have morphology similar to the MVs produced by other Gram-positive bacteria such as *Lacticaseibacillus casei* [83] and were not observed in the control MRS (Sup Fig. 2A).

We next examined the ability of MVs purified from SN *Lh* (MV *Lh*) to stimulate autophagy in host cells (Fig. 5G-J). Interestingly, treatment of cells with MV *Lh* induced a significant two-fold increase in the number of GFP-LC3 positive dots in HeLa cells (Fig. 5G-H). To validate the specificity of autophagy induction by *L. helveticus* MVs, we tested MVs from two strains, *L. acidophilus* VEL12314 and *L. paracasei* ATCC334, which were characterized in Fig. 1 as non-inducers of autophagy (Fig. 1A-E). Their MVs failed to markedly stimulate autophagy in HeLa cells (Sup Fig. 3A-B), despite being produced at levels comparable to *L. helveticus* (Sup Fig. 3C), confirming the strain-specific nature of this effect. Of note, under these experimental conditions, the pro-autophagy stimulatory effect of *L. helveticus* MVs was greater than that of the prototypical autophagy inducer, starvation (EBSS-incubated cells) (Sup Fig. 3A-B).

Finally, we also demonstrated that MV *Lh* were also able to stimulate autophagy in immune cells, as shown by a significant increase in the number of LC3 positive vacuoles in MV *Lh*-treated RAW264.7 macrophages compared to controls (Fig. 5I-J).

To explore whether the increase in GFP-LC3 positive vacuoles observed in MV *Lh*-treated cells resulted from stimulation of the autophagy process or endocytosis of MVs into LC3-positive vacuoles - a mechanism termed as “LC3-associated endocytosis” and which involves only a subset of the autophagy machinery [84] -, we labelled MV *Lh* with a lipophilic probe (DiI) to study their interactions with host cells (Sup Fig. 2B). We observed that MV *Lh* were rarely (5.6% of total MV *Lh*) found in the vicinity of LC3 positive structures (Sup Fig. 2B and 2 C), suggesting that while MV *Lh* induced autophagy, they were not extensively taken up within autophagy vacuoles.

Altogether, these results indicate that MVs produced by *L. helveticus* VEL12193 are autophagy inducers.

### Proteomic and lipidomic profiling of *L. helveticus* VEL12193 membrane vesicles identifies potential pro-autophagy-related signals

**Fig. 6:**
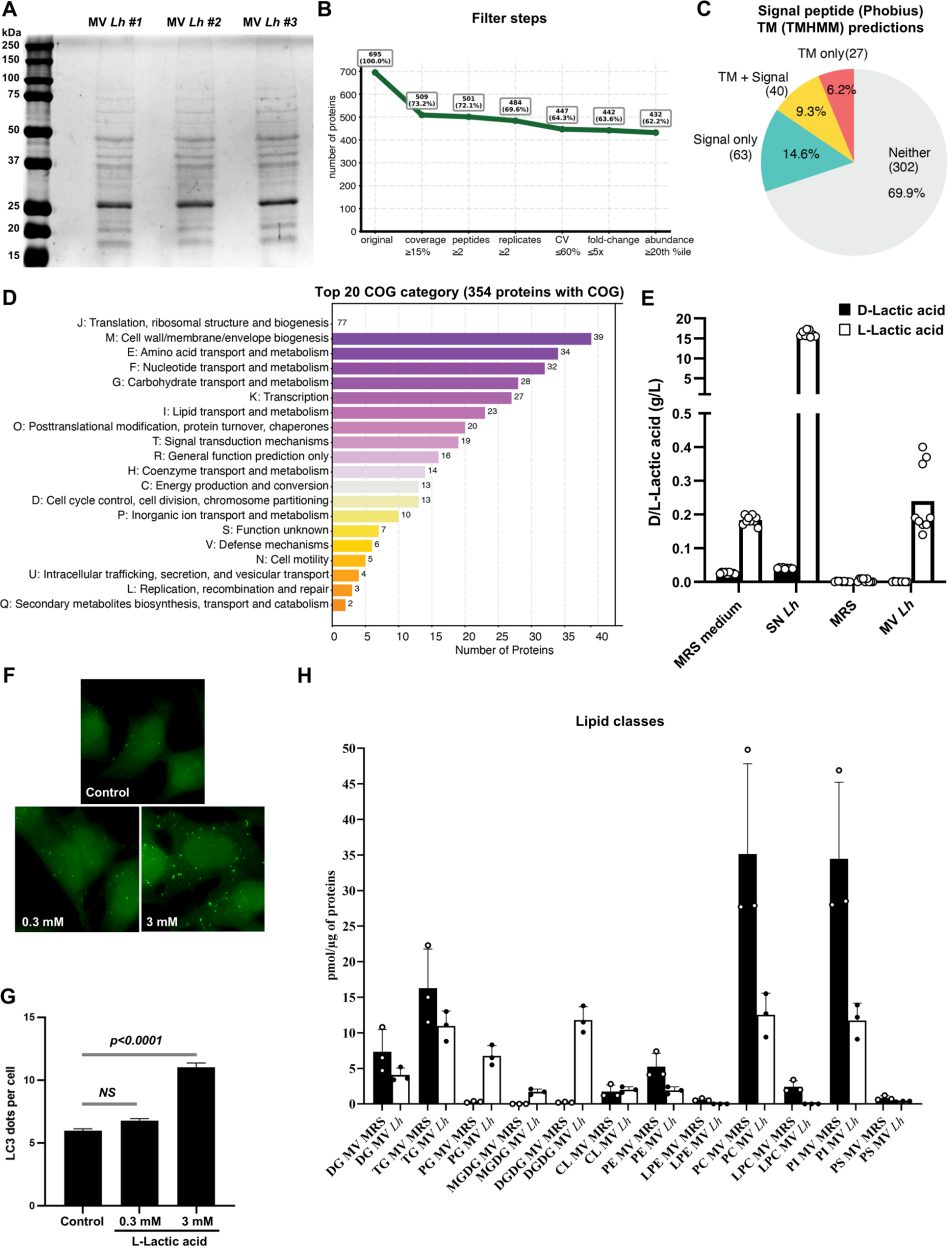
Biochemical characterization of membrane vesicles released by Lactobacillus helveticus VEL12193 and the potential role of MV-associated L-lactic acid in stimulating autophagy in epithelial cells. **A** Coomassie Blue-stained SDS-PAGE of protein extracts from MVs of L. helveticus VEL12193, representing three biological replicates (#1 to #3), illustrating the protein profile obtained. **B** Filtering pipeline to ensure high-confidence protein selection. This stringent quality control pipeline resulted in 432 high-quality protein identifications from the initial dataset of 695 proteins. **C** Domain and feature analysis highlighting signal peptide and Transmembrane segments (TM) predictions. Comprehensive protein domain and feature analysis was performed using InterProScan 5, integrating signal peptide (Phobius) and transmembrane segment (TMHMM) predictions. **D** Functional classification of filtered proteins was performed using the COG database via RPS-BLAST. High-confidence hits (E < 0.01) yielded 354 proteins assigned to 20 COG categories. The top 20 categories are displayed, category J (translation, ribosomal structure, and biogenesis) is omitted for clarity, with its count indicated separately. **E** Dosage of D- and L-lactic acids in MRS medium, L. helveticus supernatant (SN Lh), concentrated and ultracentrifugated MRS (MRS) and MVs purified from L. helveticus SN (MV Lh). **F** Representative images of GFP-LC3 HeLa cells untreated (control) or treated for 2 h with 0.3 or 3 mM of L-lactic acid. **G** Quantification of the number of LC3 dots per cell 2 h after treatment of GFP-LC3 Hela cells with L-lactic acid. Data are mean ± SEM of four independent experiments. Mann-Whitney test was used and *p*-value is indicated on the graph. **H** Lipid composition of MVs derived from L. helveticus VEL12193 and MRS medium. The lipidomics results were generated from three independent biological L.helveticus (MV Lh, white bars) and MRS medium MVs (MV MRS, black bars). For each lipid class, results are expressed as pmol of total lipids (sum of the amount of identified lipid species) per µg of protein. DG: diacylglycerols; TG: triacylglycerols; PG: phosphatidylglycerols; MGDG: monogalactosyldiacylglycerols; DGDG: digalactosyldiacylglycerols; CL: cardiolipins; PE: phosphatidylethanolamines, LPE: lysophosphatidylethanolamines; PC: phosphatidylcholines; LPC: lysophosphatidylcholines; PI: phosphatidylinositols; PS: phosphatidylserines

Proteomic and lipidomic analyses were conducted to achieve a comprehensive molecular characterization of *L. helveticus* VEL12193 MVs. SDS-PAGE analysis of protein extracts from these MVs revealed consistent protein profiles across three biological replicates (Fig. 6A). MS-based proteomic characterization of these three biological replicates of MVs allowed the reliable identification of 695 proteins (Sup. Table 1). A stringent filtering pipeline applying criteria including sequence coverage, minimum peptide count, and reproducibility across replicates was used to refine the initial dataset, resulting in 432 high-confidence protein identifications, for which comprehensive analysis of protein domains and features were performed (Fig. 6B-C). These analyses revealed that 15.5% (*n* = 67) of the MV-associated proteins are transmembrane, and 23.9% (*n* = 103) contain a signal peptide. Functional annotation of the filtered proteins was conducted using the Clusters of Orthologous Groups (COG) database through RPS-BLAST searches. High-confidence assignments (E-value < 0.01) allowed classification of 354 proteins into 20 distinct COG categories (Fig. 6D). The complete list of filtered proteins, along with their sequences and predicted functional and structural features, is provided in Sup Table 1. The identification of three lactate dehydrogenase (ldh) enzymes in the *L. helveticus* VEL12193 MV proteome (A0AAN4UJX2_LACHE, A0A6A7K2M4_LACHE and A0A3S8SDE9_LACHE) suggests that these vesicles may contain lactate. To investigate this possibility, we quantified both D- and L-lactic acids levels within the MVs. The culture supernatant of *L. helveticus* contained high levels of L-lactic acid but no detectable D-lactic acid, and L-lactic acid was also detected, at a lower level, in the MV fraction (Fig. 6E). Interestingly, L-lactic acid has been shown to stimulate autophagy [85], raising the possibility that *L. helveticus* MVs may promote host autophagy through the release of L-lactic acid within host cells. Based on the final concentration of MVs applied to host cells and their measured L-lactic acid content, we estimated that host cells were exposed to approximately 0.27 mM L-lactic acid. To assess whether this level was sufficient to elicit autophagy, HeLa GFP–LC3 cells were treated with two doses of L-lactic acid: 0.03 mM, mimicking the concentration present in *L. helveticus* MVs, and 3 mM, a dose range previously reported to induce autophagy [86]. L-lactic acid triggered a dose-dependent increase in LC3 puncta, with 0.03 mM yielding only a slight, non-significant trend toward increased dot formation, whereas 3 mM significantly elevated the number of LC3 puncta compared with control (Fig. 6F-G). These results indicate that the low L-lactic acid concentration delivered by *L. helveticus* MVs is not sufficient on its own to account for the autophagy induced by MVs, but may contribute to it.

Finally, we performed lipidomic analyses to characterize the lipid profile of *L. helveticus* VEL12193 MVs. The lipid profile obtained from the vesicle preparations following *L. helveticus* culture (MV *Lh*) was compared to that obtained from MVs preparations produced without bacterial inoculation (MRS). A summary of the concentrations of each lipid class are presented in Fig. 6H. Lipid classes enriched in *L. helveticus* MVs were PG, MGDG and DGDG (Fig. 6H). The fatty acid composition of PG, MGDG, DGDG, and CL is consistent with the fatty acid profile reported for the whole bacteria [87]. DG, TG, PE, PC and PI were present in control preparations suggesting that the source of these lipids may be the bacterial cell culture medium (MRS). Lipid species in each class and their abundances are shown in supplementary figures (Sup Fig. 4–16). Interestingly, some CL species and their direct precursors PG are enriched in *L. helveticus* MVs (Sup Fig. 4, 7–8), and these lipids are known to act as signaling molecules for recognition and targeting by autophagy, particularly in the context of selective autophagy such as mitophagy [88].

## Discussion

The autophagy machinery is crucial for maintaining homeostasis throughout life in eukaryotic organisms by modulating key cellular processes such as membrane dynamics, removal of damaged organelles, maintenance of energy balance or defense against pathogens [2, 89]. Stimulation of autophagy has been shown to be beneficial in various pathophysiological contexts and to support healthy aging [4]. Various regulatory pathways of autophagy are sensitive to microorganisms and their metabolites, as evidenced by the modulatory effects of gut microbiota on host autophagy [27]. In this study, we explored the potential of food grade bacteria and bacteria belonging to species used as probiotics to stimulate autophagy by combining in vitro and in vivo approaches.

A growing number of studies have described the ability of specific strains from the lactobacilli family or the *Bifidobacterium* genus to modulate host autophagy in vitro [90–92]. In this study, we showed that not all strains of lactobacilli and bifidobacteria were able to stimulate autophagy and that it is important to verify this phenotype by different complementary approaches. Indeed, among the 11 strains we tested, only one showed a robust autophagy-stimulating capacity that was validated by the different approaches used. To date, only a few studies have analyzed the ability in vivo of beneficial microorganisms to modulate autophagy in mammals [90, 93–95]. Indeed, although many autophagy-related molecular markers exist, it remains highly challenging to properly assess autophagy responses in vivo, notably due to the highly dynamic nature of this process [96]. In addition, in vivo analysis of autophagy is usually limited to the measurement of the basal expression level of ATG proteins or genes, which are parameters that do not fully inform about the dynamic state of this process. In the present study, we validated the in vivo pro-autophagic property of *L. helveticus* VEL12193 by combining again different approaches including a study of autophagy flux on tissues ex vivo. We chose to work ex vivo rather than in vivo - which is possible by administrating autophagy inhibitors to mice - for several reasons. Firstly, by carrying out the autophagic flux analysis ex vivo, we limited the variability associated with the use of two different animals (one for the analysis of autophagic flux when “open” (without the use of autophagic flux inhibitors) and the other for the analysis of autophagic flux when “closed” (after injection of autophagic flux inhibitors)). Indeed, in the ex vivo experiment, each animal constitutes its own control. Secondly, ex vivo experimentation overcomes the problem of bioavailability of autophagy inhibitors to tissues/organs, which may be limited by biological barriers. Finally, ex vivo analysis of autophagy flux enables also to limit the number of mice used in the experiment (each animal being its own control). Of note, some tools are available to study autophagy flux in tiny model organisms such as in *Caenorhabditis elegans* or in Zebrafish [97, 98].

The prototypical ATG protein LC3 is widely used as a gold standard autophagy marker since its conjugation to membrane forming autophagosome is a key event during canonical autophagy. However, this protein can also be conjugated to other cellular membranes, a recently described process termed ATG8ylation [99]. ATG8ylation involved some ATG proteins of canonical autophagy such as ATG16L1 but not all. In particular, WIPI2 and ATG13 are not involved. Colocalization of WIPI2 protein with LC3 puncta in cells treated with *L. helveticus* suggests a stimulation of the canonical form of autophagy. In addition, the observation of an increase in the number of LC3 puncta in cells treated with *L. helveticus* in the presence of Bafilomycin A1, a drug known to inhibit non-canonical forms of autophagy [100], supports the stimulation of canonical autophagy by *L. helveticus* (data not show). However, it is important to remain cautious in drawing conclusions and not to exclude the possibility of non-canonical forms of autophagy, which may involve only parts of the autophagy machinery without the formation of canonical double-membraned autophagosomes. The use of cells deficient for key ATGs genes (ULK1, ATG9 and ATG13) involved in canonical autophagy but dispensable for non-canonical forms of autophagy might be useful for go further.

Selective autophagy corresponds to the lysosomal degradation of specific intracellular components (e.g., proteins aggregate (aggrephagy), mitochondria (mitophagy), intracellular bacteria (xenophagy), peroxisome (pexophagy)). This involves activity of selective autophagy receptors (SARs) which interact with ATG8 family proteins [101]. Impairment of certain forms of selective autophagy has been associated with the development of pathophysiological conditions such as xenophagy in IBD such as CD, and mitophagy and/or aggrephagy in age-related diseases such as AMD and Alzheimer’s disease [102]. Thus, it might be of interest to go further in the analysis of pro-autophagic property of *L. helveticus* and its related MVs by analyzing selective forms of autophagy, which can be done by using specific markers of these processes [101]. Interestingly, the yeast *Saccharomyces boulardii* strain CNCM-I-1079 and the bacteria *Lactococcus lactis* strain R1058 have been shown to stimulate mitophagy in vitro and in vivo in Drosophila model, thus leading to neuroprotective effects [90].

Even if a growing number of studies point out the ability of some beneficial microbes to stimulate autophagy, to our knowledge, no study has definitively identified the specific microbial compound(s) responsible for this pro-autophagic property. Bacterial supernatants from strains such as *Levilactobacillus brevis*, *Lactiplantibacillus plantarum*, *Lacticaseibacillus rhamnosus* and *Lacticaseibacillus paracasei*, has been identifed as the active fraction having pro-autophagy property [93, 103–106]. In our study, we identified the MVs released in the supernatant of *L. helveticus* as an active compound able to stimulate autophagy. It would be interesting to analyze whether MVs are also responsible for the supernatant-induced stimulation of autophagy described for other strains. Unlike Gram-negative bacteria, in which MV formation occurs directly through outer membrane blebbing, resulting in outer-membrane vesicles (OMVs), MV formation in Gram-positive bacteria, such as *L. helveticus*, requires local or global (bubbling cell death) peptidoglycan degradation, allowing the cytoplasmic membrane to protrude through holes in the peptidoglycan layer [107]. In comparison to the production of MV by Gram-negative bacteria, much less is known regarding the regulation of MV production by Gram-positive bacteria. Several stress such as treatment with antibiotics (β-lactam family), DNA damaging agents or peptidoglycan degrading enzymes produced by other microorganisms that weaken the bacterial cell wall, have been identified to promote MV production in Gram-positive bacteria [83, 108]. Physiological stimuli favoring MV production in Gram-positive bacteria, particularly in the context of the gastrointestinal tract environment, remain to be determined. It would be particularly interesting to investigate the in vivo production of these MVs in the gastrointestinal tract, where they can interact with intestinal epithelial cells and potentially diffuse to peripheral organs, as suggested for some Gram-negative bacteria of the gut microbiota [109].

Analyses of MVs released by Gram-positive bacteria have shown a highly complex composition that includes lipids (e.g., phospholipids or lipoteichoic acid), hundreds of proteins, nucleic acids and other metabolites [108]. This complexity makes it challenging to pinpoint the specific compound(s) responsible for an effect, in our case stimulation of autophagy in host cells. DNA, RNA and peptidoglycan of MVs derived from *Staphylococcus aureus* have been reported to activate Toll-like receptor (TLR) 2, 7, 8 and 9 as well as the intracellular Nucleotide-binding Oligomerization Domain 2 (NOD2) receptor in lung epithelial cells [110]. Lipoteichoic acids (LTAs) or lipoproteins from lactobacilli have been shown to be present on MVs and to act as TLR2 ligands [111, 112]. Six lipoproteins (A0AAJ3YM52_LACHE, A0A386RGR0_LACHE, A0A0R2M2W0_LACHE, A0AAJ3YKK9_LACHE, A0A0R2MK76_LACHE, and A0A2 × 0R847_LACHE) were found associated with the MV fraction in our proteomic analysis and could potentially act as TLR2 ligands. Although LTAs themselves cannot be detected by proteomic analysis, several proteins involved in their biosynthesis (e.g. LtaS2, DltA, DltC, and DltD) were identified in the MV proteome, suggesting that LTAs might be present in the MV fraction and could contribute to the stimulatory effects of *L. helveticus* MVs on autophagy. Activation of these receptors, alone or in combination, have been shown to modulate autophagy in various cell types [24, 110]. These findings suggest that such bacterial molecular patterns and host receptors could also contribute to autophagy stimulation by *L. helveticus* strain VEL12193 [113]. Beyond PRRs ligands, small organic molecules produced by lactobacilli, such as lactate, can be found in MVs [114] and represent also autophagy inducers [85, 86]. In line with this hypothesis, we identified three lactate dehydrogenase (ldh) enzymes in the MV proteome and detected lactate in the MV-enriched fraction. However, although the concentration of lactate found in *L. helveticus* MVs can slightly induce autophagy, it is not sufficient on its own, suggesting that other MV-associated factors are required to elicit the full autophagic response. Our lipidomic analysis also suggest that the lipid composition of *L. helveticus* MVs may contribute to their capacity to stimulate autophagy. The presence of CL species and their precursor PG within the vesicles is particularly noteworthy, as these lipids function as signals for autophagic targeting, particularly in selective pathways such as mitophagy [88]. *L. helveticus* MVs also contain PE and PS, which have been reported to stimulate autophagy [115, 116], however, based on our analysis, we cannot exclude the possibility that these lipid classes originate from the bacterial culture medium rather than from the MVs themselves.

It has been proposed that OMVs released by Gram-negative bacteria can be internalized in non-phagocytic host cells through several routes including clathrin- or caveolin-mediated endocytosis, micropinocytosis or lipid rafts [117]. Fusion events of MVs from Gram-positive pathogens such as *S. aureus* or *Listeria monocytogenes* with host cells have also been suggested, enabling the transfer of virulence factors (α-hemolysin and listeriolysin O, respectively) to the host plasma membrane [118, 119]. Much less is known about the trafficking of MVs from beneficial bacteria within host cell. Endocytosis of MVs released by *Lacticaseibacillus rhamnosus –* a bacterium having immunomodulatory effects - has been described in vitro in intestinal epithelial cells [120]. We observed that *L. helveticus* MVs were tightly associated with host cells and that some of them colocalized within LC3 positive structures, offering the intriguing possibility that internalized bacterial MVs can be handled into endomembrane connected to the autophagy pathway. This has already been suggested for OMVs released by the Gram-negative bacteria *Helicobacter pylori* and *Pseudomonas aeruginosa* that colocalized with endosomes, in close association with LC3-positive vacuoles [121]. Similarly, *S. aureus*-derived MVs were shown to colocalize with LC3-positive structures (110). Further investigations are needed to better understand the molecular interactions between *L. helveticus* MVs and the autophagy machinery.

The *L. helveticus* strain VEL12193, identified in this study among 11 strains tested of lactobacilli and bifidobacteria as the best candidate to stimulate autophagy in host cells, has been isolated from a fermented food, a French cheese (Comté). Fermented foods therefore appear to represent a rich source for discovering microorganisms with significant potential benefits for human health. Indeed, there are an estimated 7,500 species of fungi and bacteria associated with fermented foods, each comprising many different strains [122]. The abundance and bioavailability of the bioactive metabolites produced by these microorganisms would depend on their local environment (e.g., plant, food matrix or digestive tract). Food matrices can modify probiotic functionalities in term of survival in the gastrointestinal tract or ability to colonize the gut mucosa [123]. For instance, some lactic acid bacteria isolated from traditional fermented foods (cheese, kimchi, or fermented soybean) can produce gamma-aminobutyric acid (GABA), an amino acid having multiple physiological functions, including autophagy stimulation in macrophages and its associated antimicrobial response [124]. Interestingly, GABA production is both strain- and food-matrices-dependent [125]. Indeed, its synthesis is controlled by the glutamate decarboxylase (GAD), and it is highly dependent on the presence of L-glutamic acid -its precursor -and pyridoxal 5′-phosphate -a cofactor- in the bacterial microenvironment. Moreover, production of bioactive molecules by microorganisms can also be modulated by diet. In a human trial, consumption of a plant-based diet for 5 days was sufficient to induce a two-fold increase in the production of the SCFA acetate and butyrate by the gut microbiota in comparison to individuals consuming an animal-based diet [126]. Given the important role of SCFA in the regulation of autophagy [27], we can hypothesize that such diet-induced changes in SCFA abundance can potentially impact autophagy activities in host cells.

## Conclusions

In this study, we highlight the potential of the food-grade bacterium *L. helveticus* and its derived products (MVs) to promote autophagy in vitro, with evidence of autophagy induction by the bacteria in vivo both locally at the gut mucosa and in distant tissues. Our results provide a proof-of-concept for developing microbial-based functional foods aimed at promoting autophagy, although the effects of fermented products containing the strain have yet to be evaluated. Further research is needed (i) to confirm whether MVs stimulate autophagy in vivo, both locally in the gut and in distant organs (ii) to identify more accurately the active molecule(s) of MVs from *L. helveticus* that are autophagy inducers and/or (iii) to understand the molecular mechanisms that lead to stimulation of autophagy by MVs. Importantly, it will also be necessary to evaluate the functional consequences of retinal autophagy modulation by *L. helveticus* to fully establish its therapeutic potential and benefits in aging and AMD. Building on this, our study highlights the promise of bacterial MVs as postbiotics and supports further research into their application for preventing diseases associated with autophagy dysfunction (e.g., age-related diseases such as AMD) as well as conditions that may benefit from enhanced autophagy (e.g., infections).Compared with live bacteria, MVs can exhibit greater stability, safety, and biocompatibility, positioning them as promising postbiotic candidates for the treatment of specific diseases [127]. Their ability to deliver bioactive molecules without the risks associated with live microbial administration further underscores their translational therapeutic potential. Notably, a meningococcal group B vaccine incorporating MVs from *Neisseria meningitidis* is already commercially available, demonstrating the feasibility and safety of MV-based biomedical applications [128]. However, non-vaccine therapeutic uses of microbial MVs are still confined to preclinical studies, where they have shown promising results in various animal models [129].

## Supplementary Information

Supplementary Material 1.

Supplementary Material 2.

Supplementary Material 3.

## Data Availability

The data that support the findings of this study are available from the corresponding authors upon reasonable request.
