# Supplementary material for: The food grade bacterium Lactobacillus helveticus VEL12193 promotes autophagy by releasing membrane vesicles
Source: Cell Commun Signal. 2026 Jan 6;24:85. doi: 10.1186/s12964-025-02616-y (PMC12871000; doi:10.1186/s12964-025-02616-y)

**Figure 1D LC3 blot**

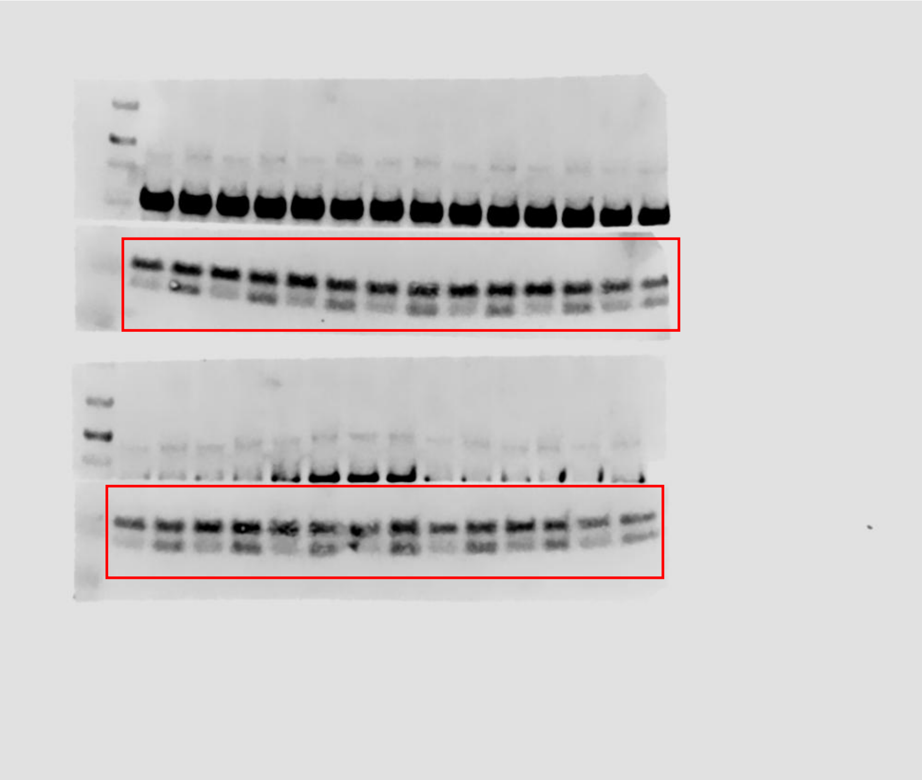

**Figure 1D Actin blot**

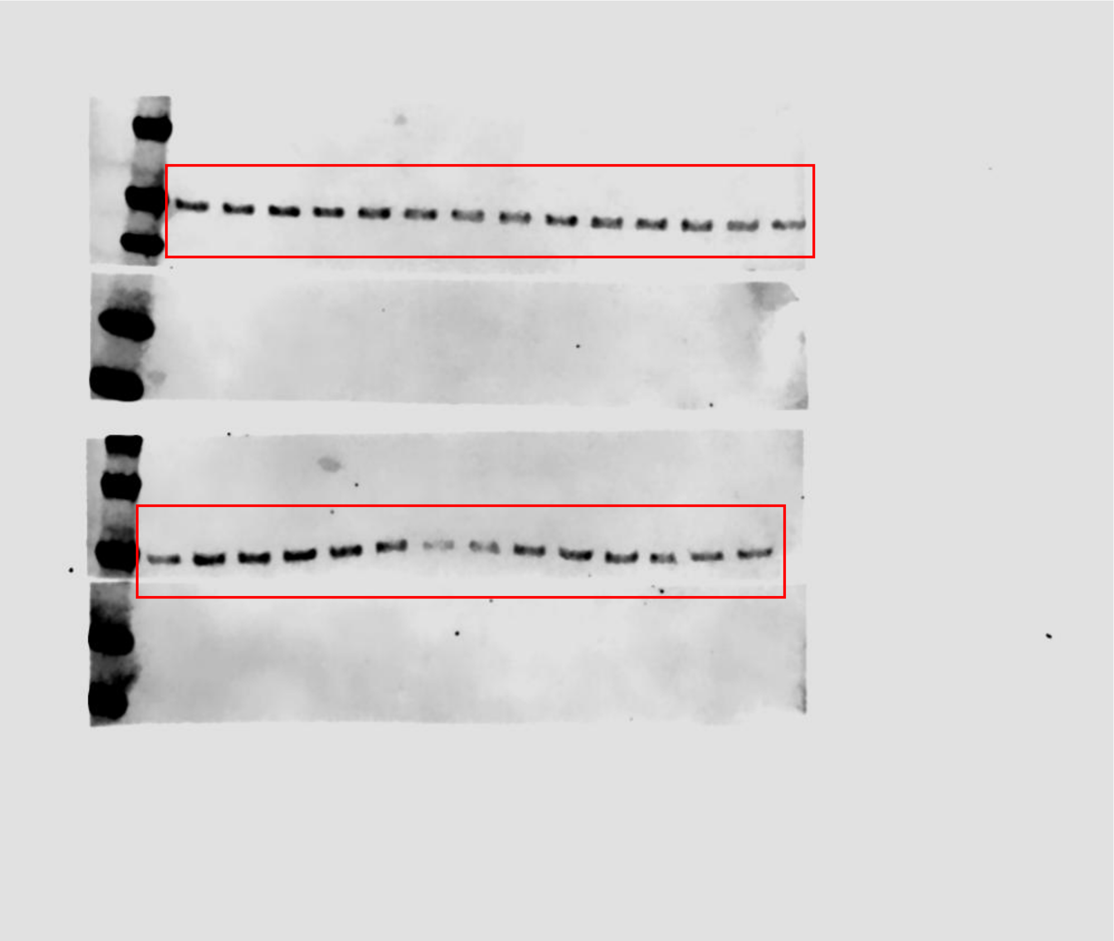

**Figure 2D LC3 blot**

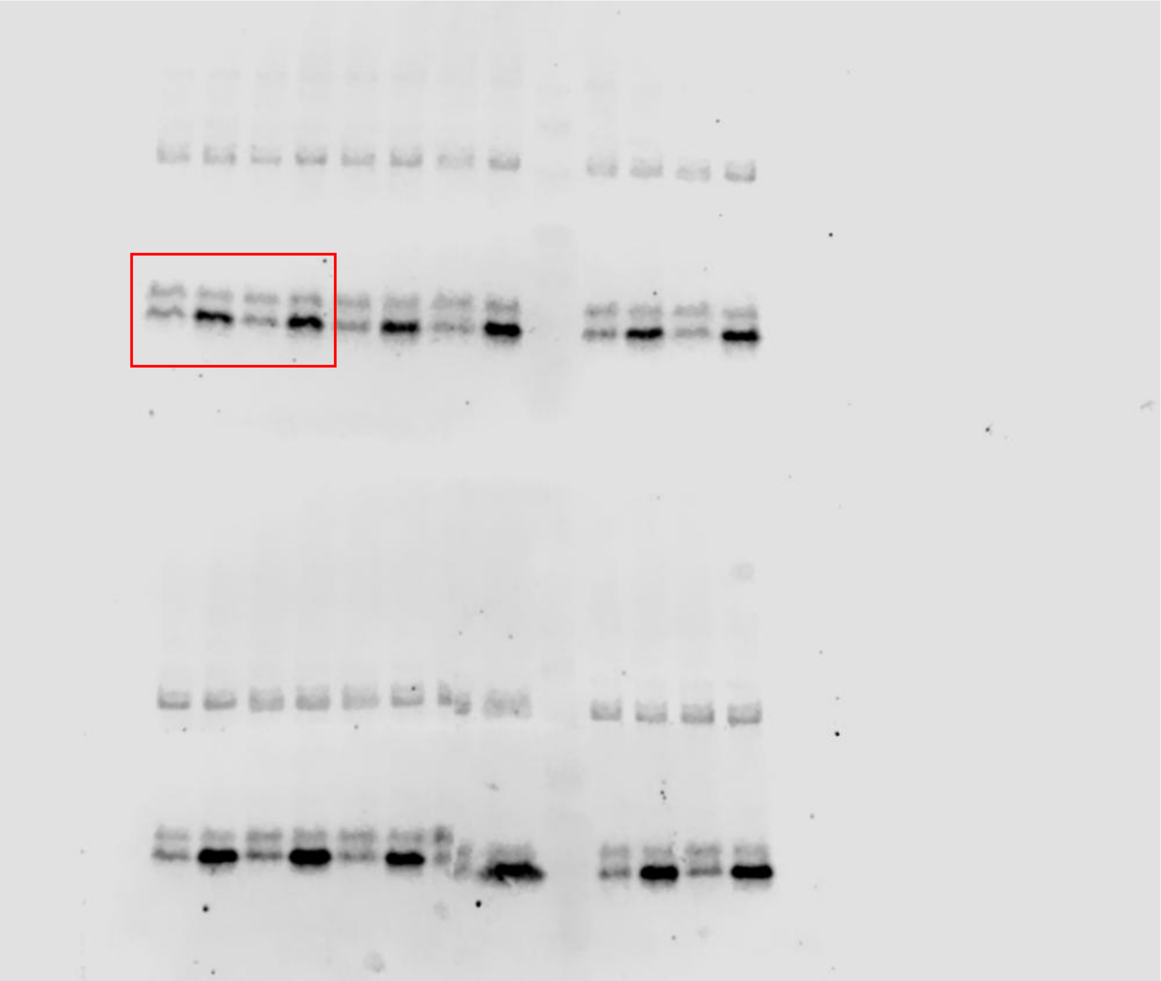

**Figure 2D Actin blot**

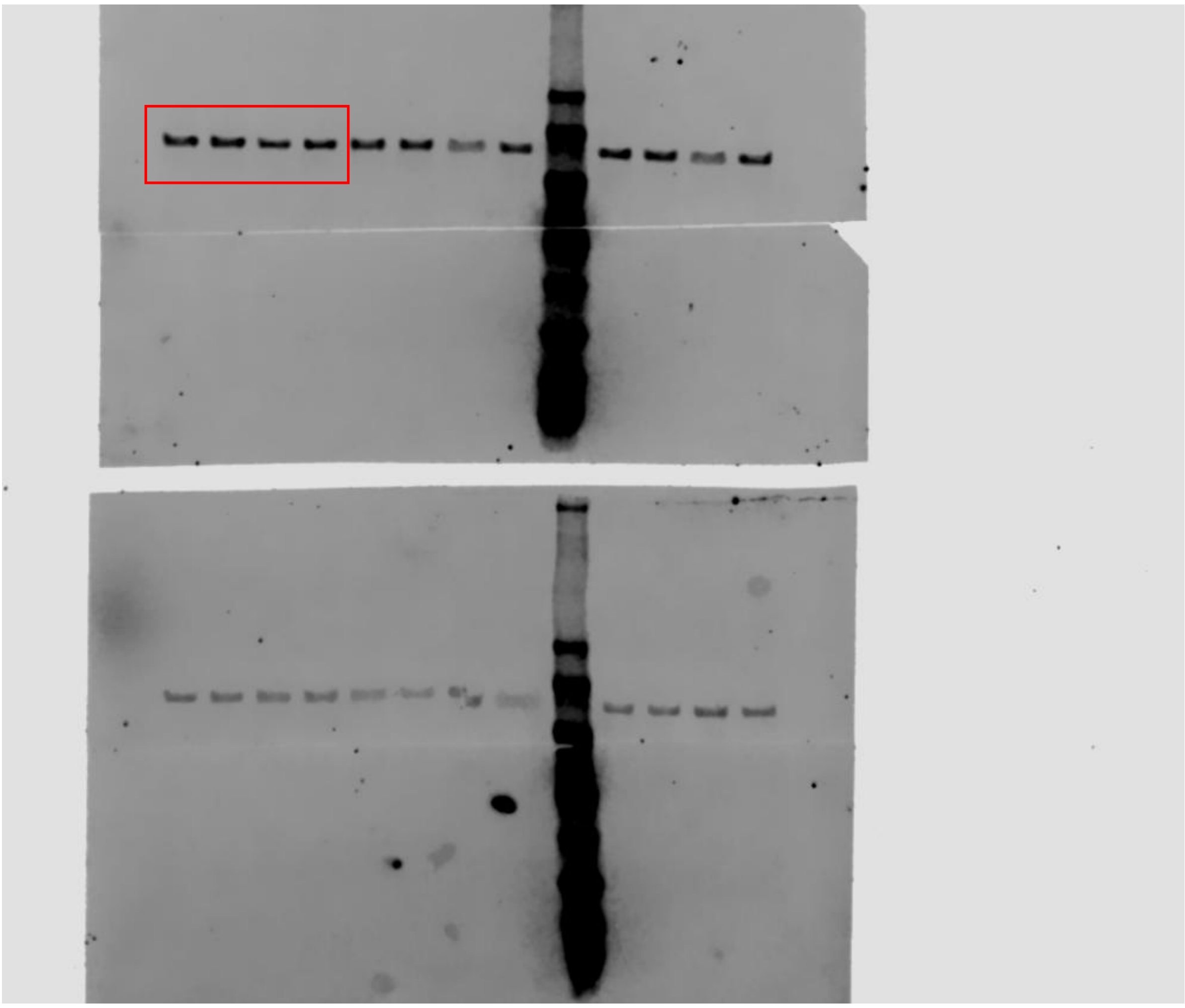

**Figure 3A LC3**

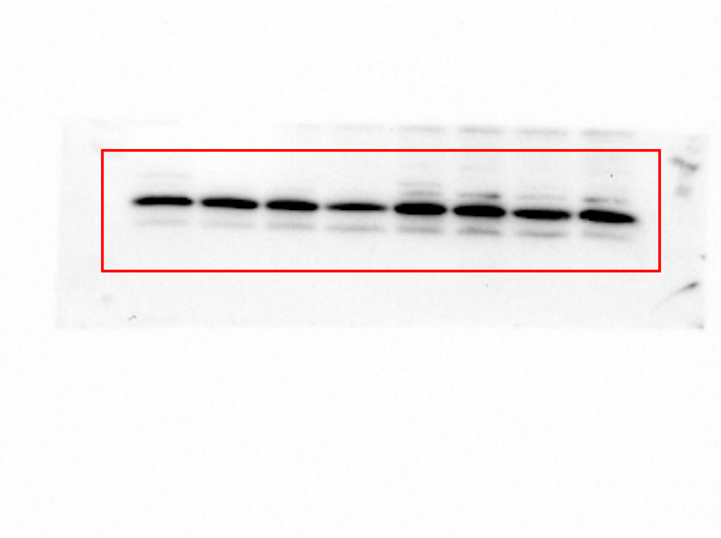

**Figure 3A p62**

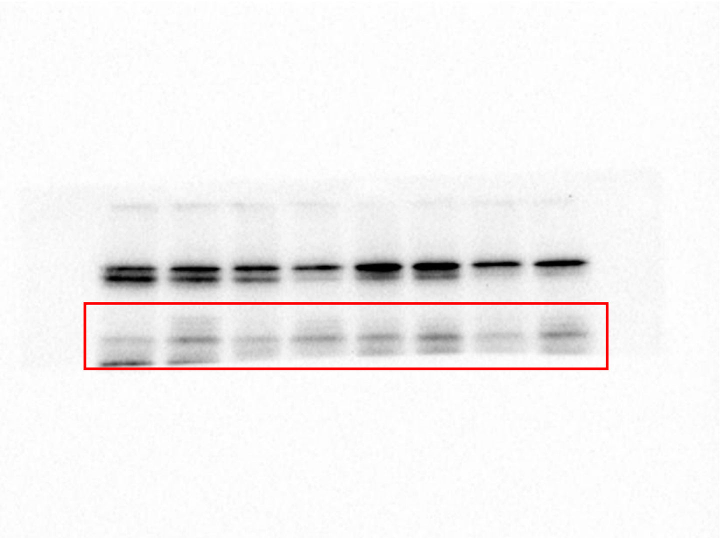

**Figure 3A Actin**

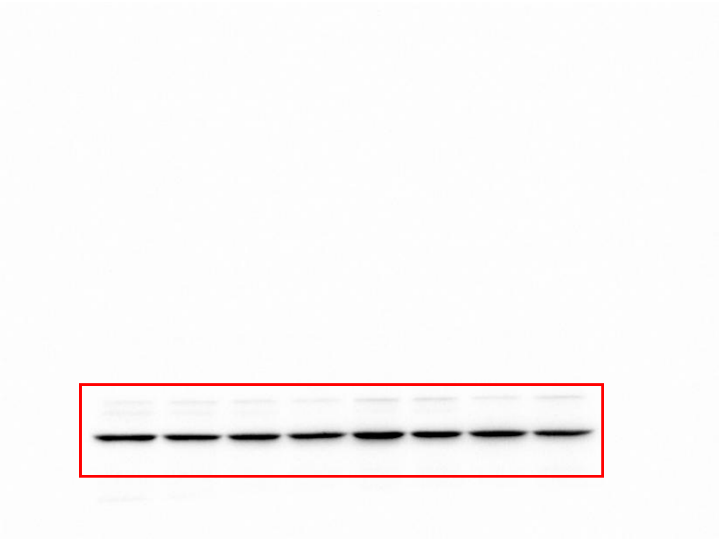

**Figure 4A LC3**

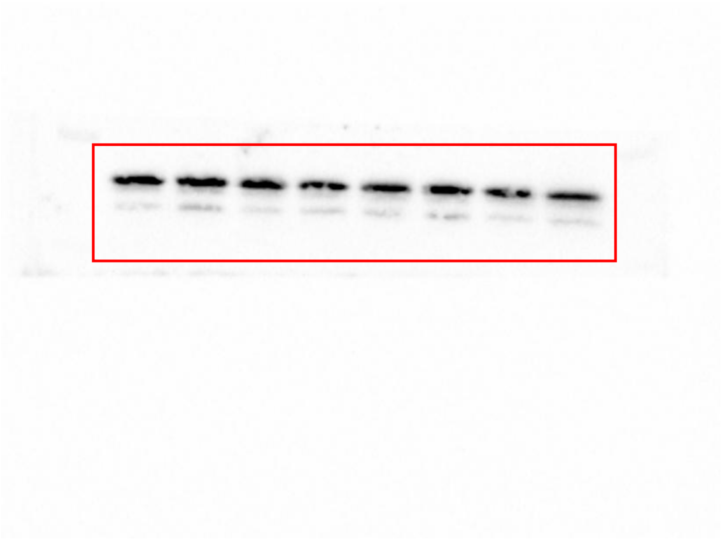

**Figure 4A p62**

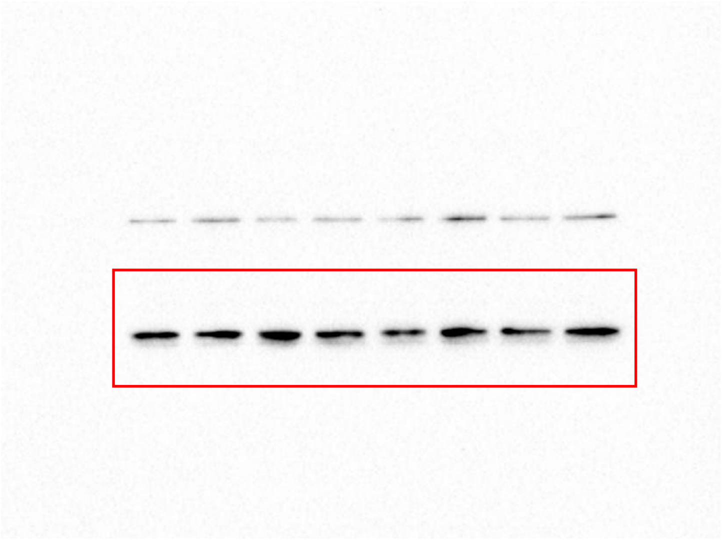

**Figure 4A LAMP2a**

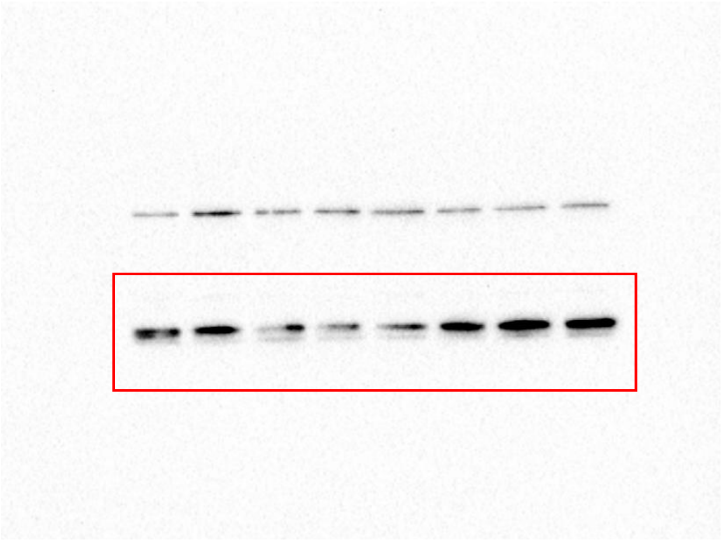

**Figure 4A Actin**

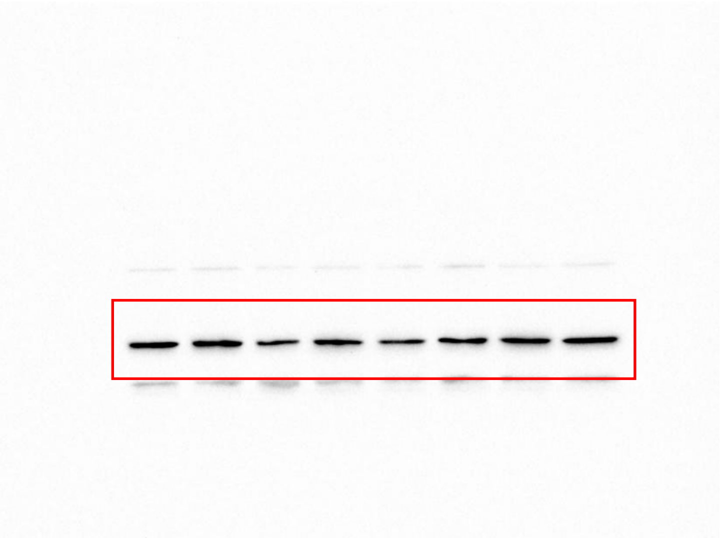

**Figure 5C LC3**

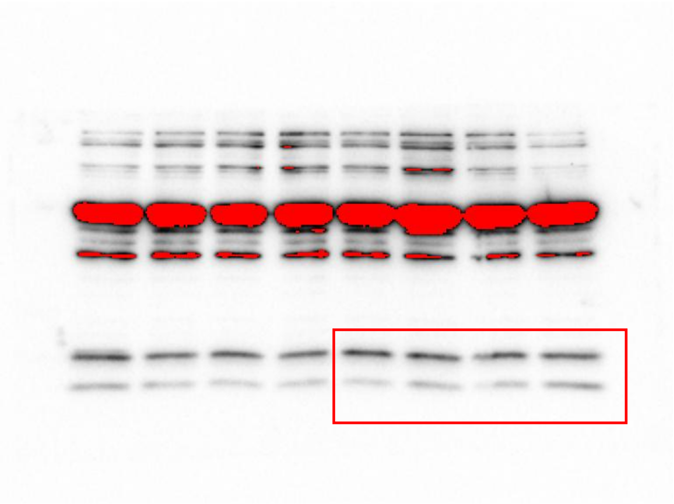

**Figure 5C Actin**

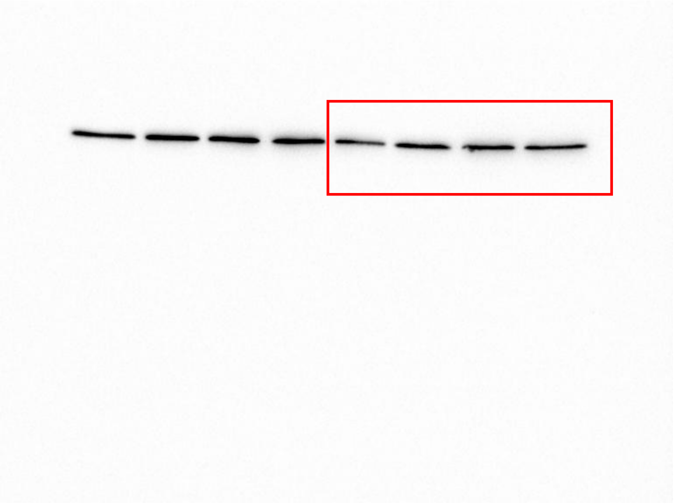

Supplement: Supplementary file 1 — Supplementary Material 1. [file 12964_2025_2616_MOESM1_ESM.pdf]
