# Supplementary material for: The food grade bacterium Lactobacillus helveticus VEL12193 promotes autophagy by releasing membrane vesicles
Source: Cell Commun Signal. 2026 Jan 6;24:85. doi: 10.1186/s12964-025-02616-y (PMC12871000; doi:10.1186/s12964-025-02616-y)
Supplement: Supplementary file 2 — Supplementary Material 2. [file 12964_2025_2616_MOESM2_ESM.docx]

**Supplementary figures**

***
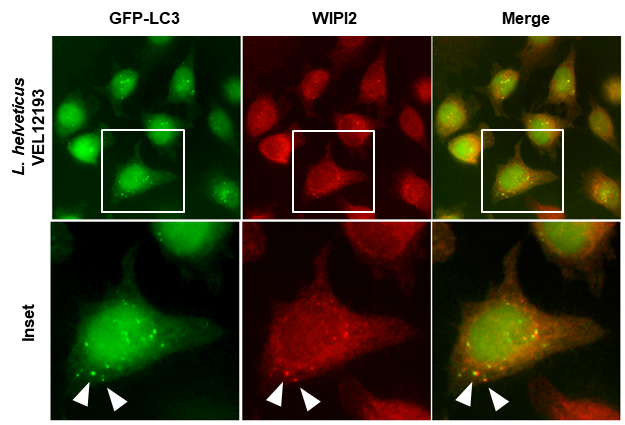
***

***Supplementary Figure 1. Colocalization of LC3 and WIPI2 in L. helveticus-treated GFP-LC3 Hela cells.***

Representative images of GFP-LC3 HeLa cells treated for 2 h with *L. helveticus* VEL12193. Cells were immunostained with anti-WIPI2 antibody (red). The GFP-coupled LC3 protein (GFP-LC3) appears in green. White squares in upper panels indicate inset areas displayed in the corresponding lower panels. Arrows indicate colocalization of LC3 and WIPI2 dots.

***
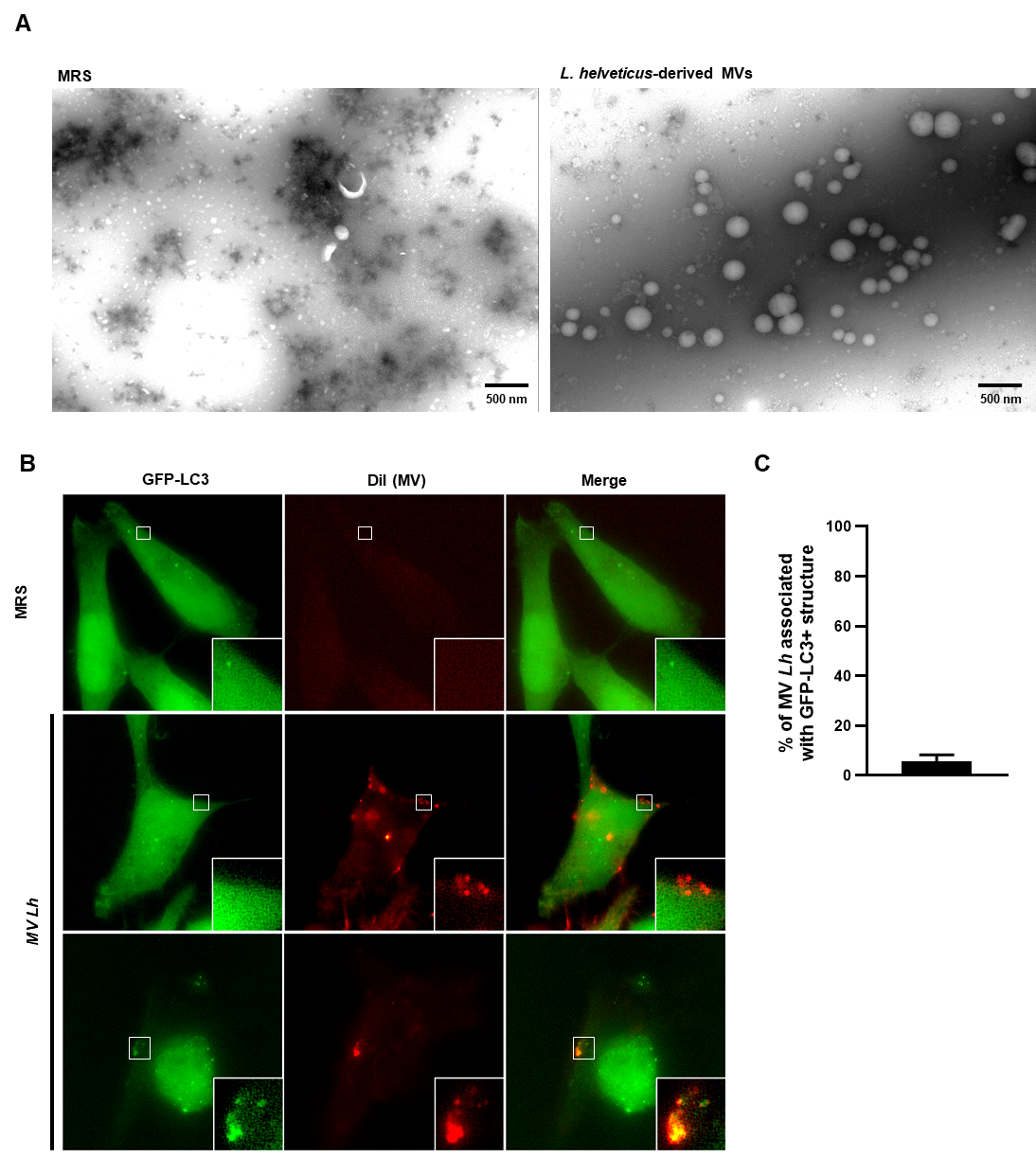
Supplementary Figure 2. Microscopic analyses of membrane vesicles released by L. helveticus VEL12193.***

(**A**) Negative-staining transmission electron microscopy image of purified MV fractions obtained from bacterial cell culture medium (MRS medium, control) or SN of *L. helveticus* VEL12193. (**B**) Representative images of GFP-LC3 HeLa cells treated for 6 h with Vybrant DiI-labelled MV fractions purified from MRS medium (control) or cell-free SN of *L. helveticus* VEL12193. MVs labelled with DiI are in red and GFP-coupled LC3 protein (GFP-LC3) appears in green. White squares indicate inset areas. (**C**) Percentage of *L. helveticus*-derived MVs (DiI-positive) co-localizing with GFP-LC3 dots.


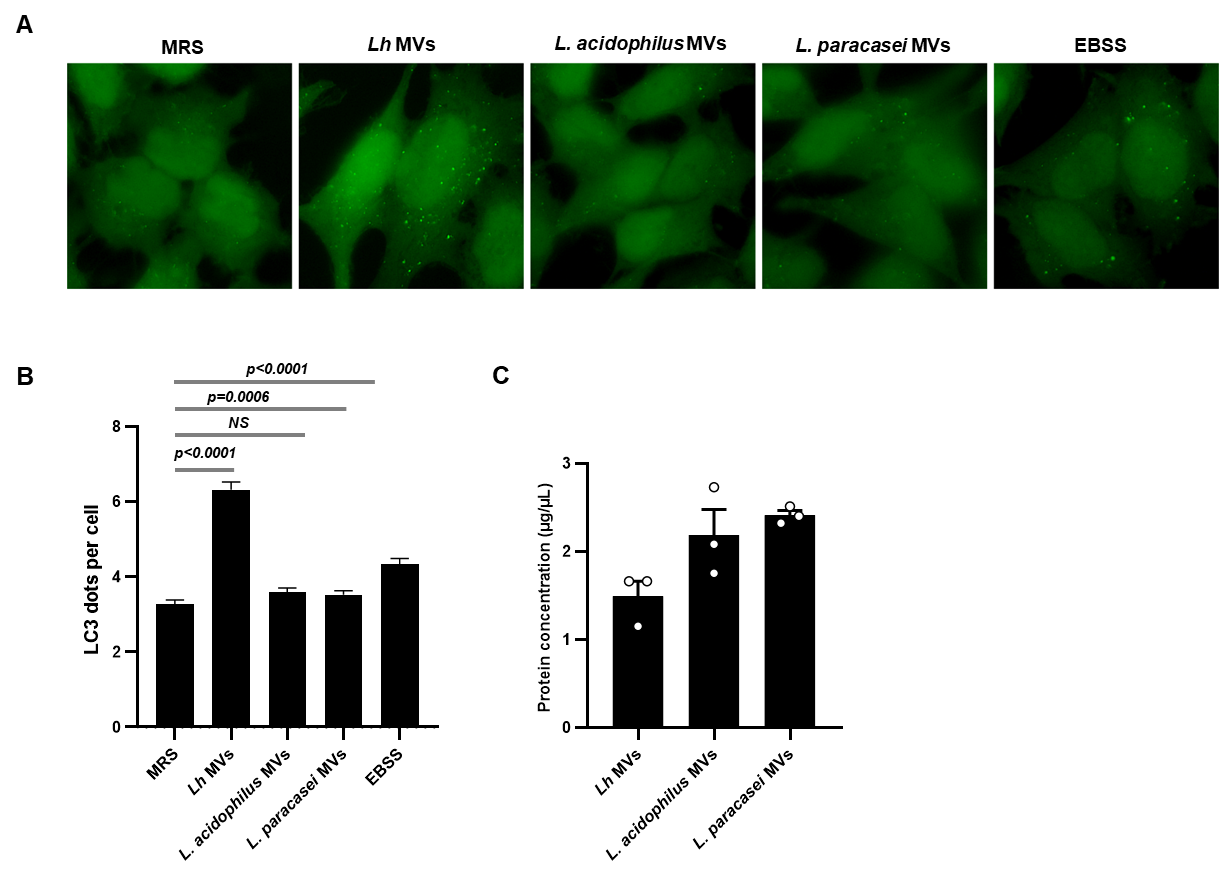


***Supplementary Figure 3. Comparison of the autophagy-stimulating properties of MVs released by L. helveticus VEL12193, L. acidophilusVEL12314 and L. paracasei ATCC334 and starvation.***

(**A**) Representative images of GFP-LC3 HeLa cells treated for 4 h with MVs purified from *L. helveticus* SN (*Lh* MVs), *L. acidophilus* SN (*L. acidophilus* MVs), *L. paracasei* SN (*L. paracasei* MVs), or MRS medium (MRS). The GFP-coupled LC3 protein appears in green. (**B**) Quantification of the number of LC3 dots per cell 4 h after treatment of GFP-LC3 Hela cells with MVs or starvation. Data are mean +/- SEM of three independent experiments, with at least 100 cells counted per condition. Mann-Whitney test was used and p-value is indicated on the graph. (**C**) Assessment of MVs quantity based on protein concentration in the different MVs fractions. Results are expressed in µg/µL. Data are mean +/- SEM of three independent experiments.

***Supplementary Figure 4. Phosphatidylglycerols (PG) species in L. helveticus VEL1293 MVs.*** *Results are expressed as pmol per µg of proteins.* With the exception of PG (16:0/18:1) and PG (16:1/18:1), PG were present only in *L.helveticus* MVs. Notably, the major forms of PG were derived from C14:0, C16:0, C16:1, C18:1, and Cyc 19:0 esterified fatty acids.


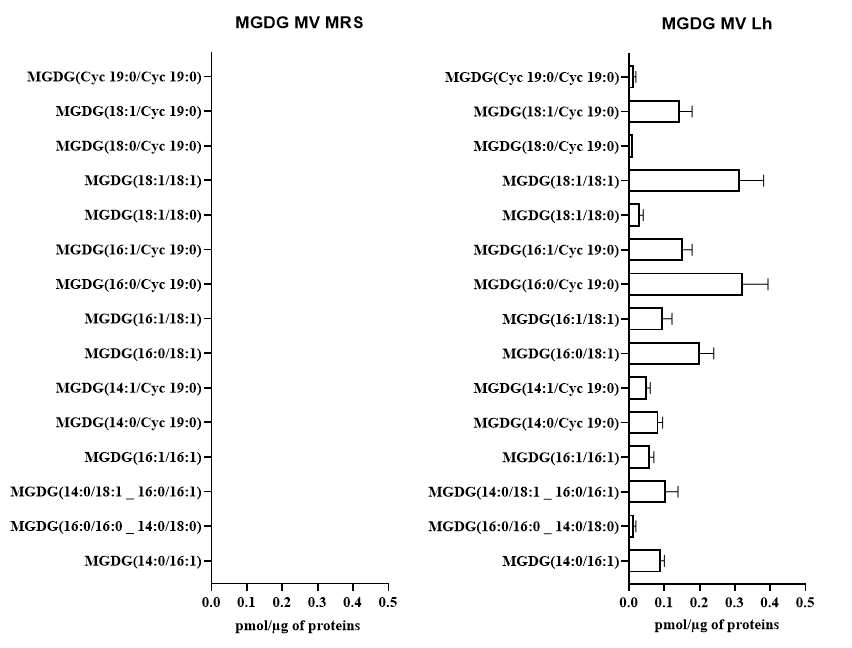


***Supplementary Figure 5. Monogalactosyldiacylglycerols (MGDG) species in L. helveticus VEL12193 MVs.*** Results are expressed as pmol per µg of protein.

***
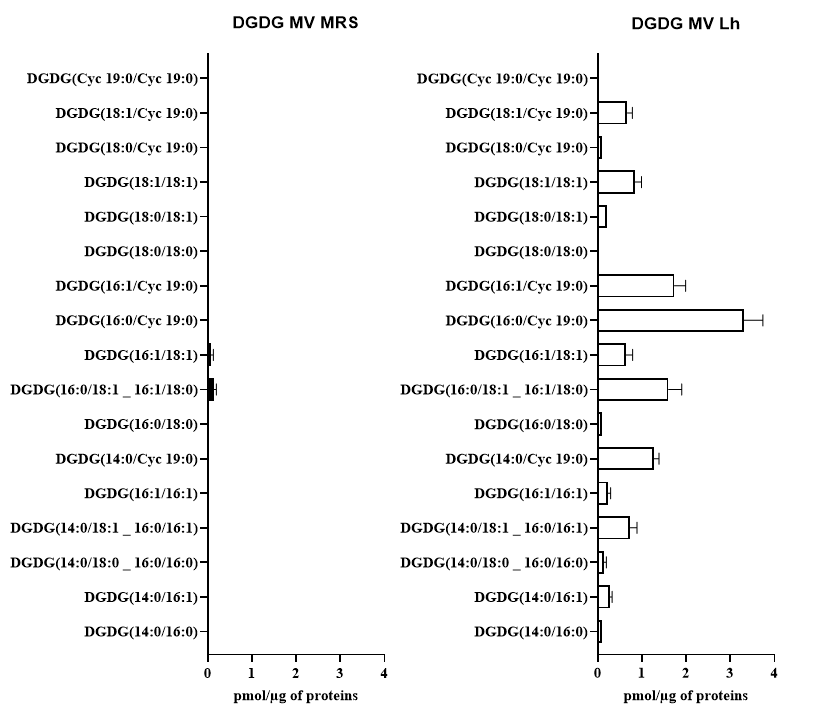
***

***Supplementary Figure 6. Digalactosyldiacylglycerols (DGDG) species in L. helveticus VEL12193 MVs.*** Results are expressed as pmol per µg of protein.


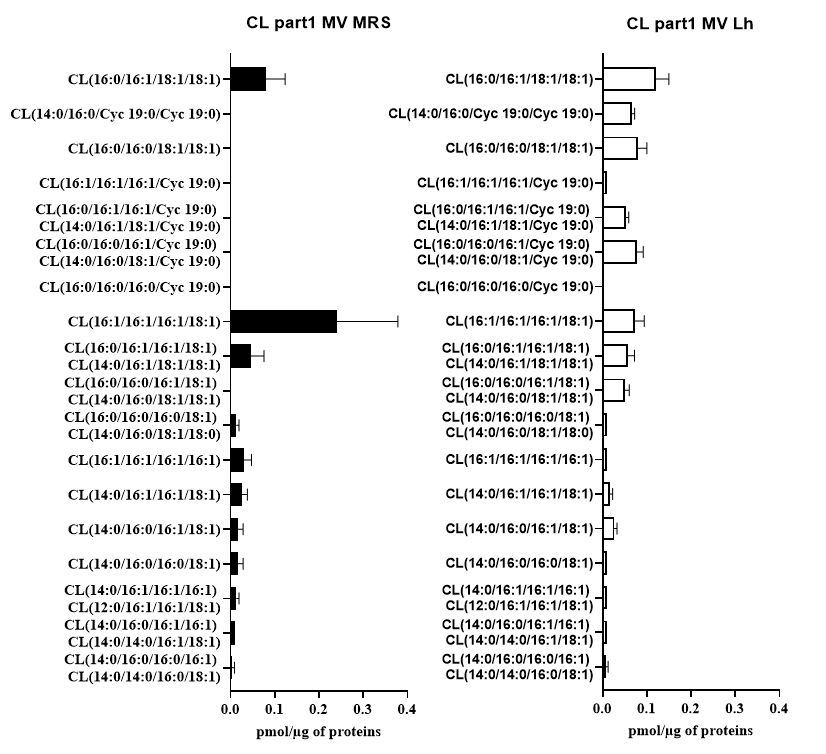


***Supplementary Figure 7. Cardiolipin (CL) species (part 1) in L. helveticus VEL12193 MVs.*** Results are expressed as pmol per µg of protein.

***
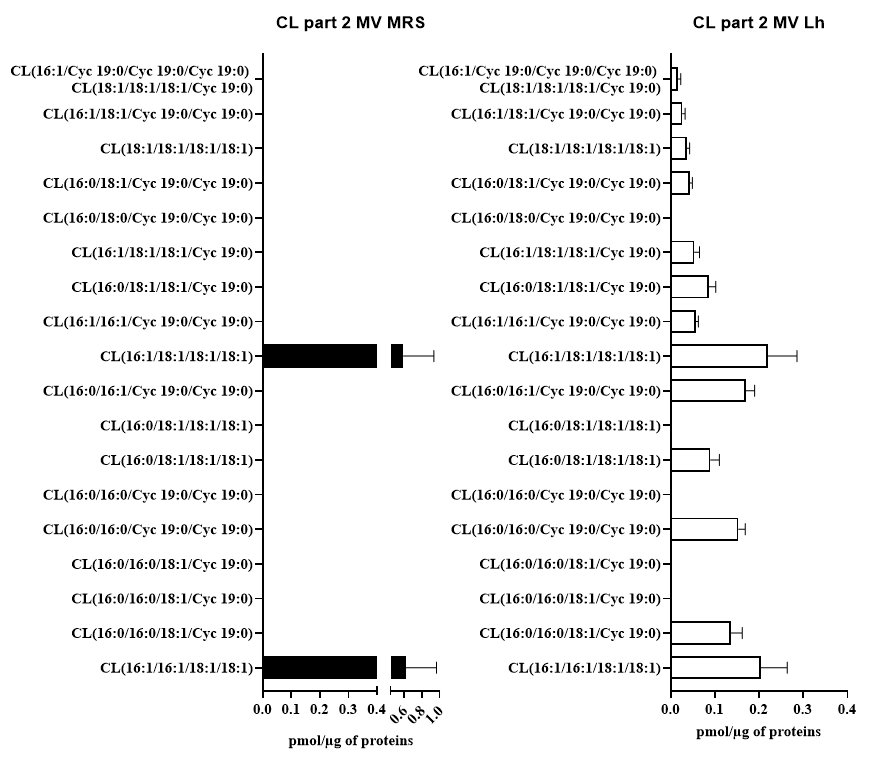
***

***Supplementary Figure 8. Cardiolipin (CL) species (part 2) in L. helveticus VEL12193 MVs.*** Results are expressed as pmol per µg of protein.

***
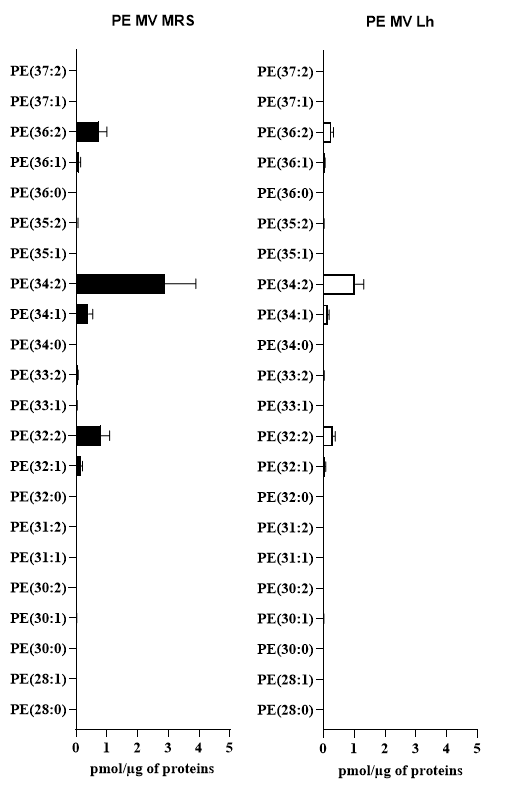
***

***Supplementary Figure 9. Phosphatidylethanolamines (PE) species in L. helveticus VEL12193 MVs.*** Results are expressed as pmol per µg of protein.

***
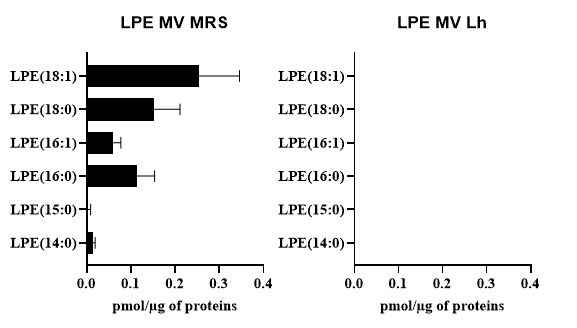
***

***Supplementary Figure 10. Lysophosphatidylethanolamine (LPE) species in L. helveticus VEL12193 MVs.*** Results are expressed as pmol per µg of protein.

***
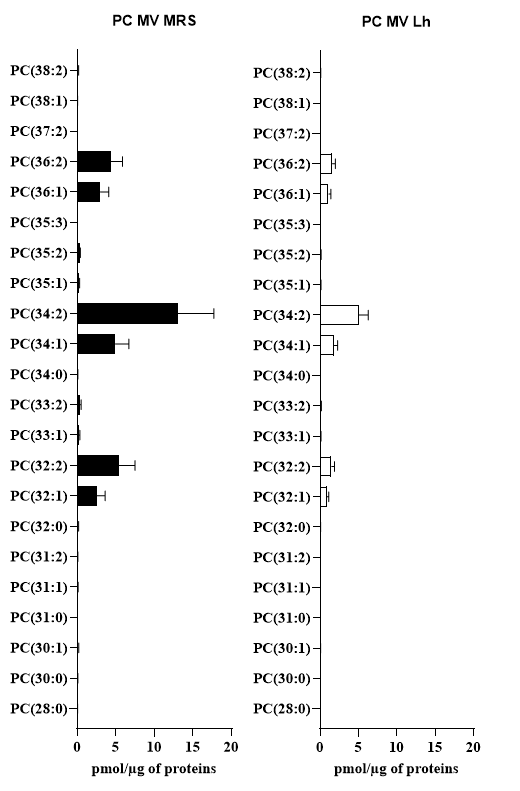
***

***Supplementary Figure 11. Phosphatidylcholines (PC) species in L. helveticus VEL12193 MVs.*** Results are expressed as pmol per µg of protein. PC, the major lipid class in MRS media membrane, was also present in *L.helveticus* MVs. Uneven carbon chain lipid species (e.g PC 33:1 and PC 35:1) were however not detected in *L.helveticus* MVs. This also implies lack of Cyc 19:0 esterified PC lipid species in *L.helveticus* MVs.

***
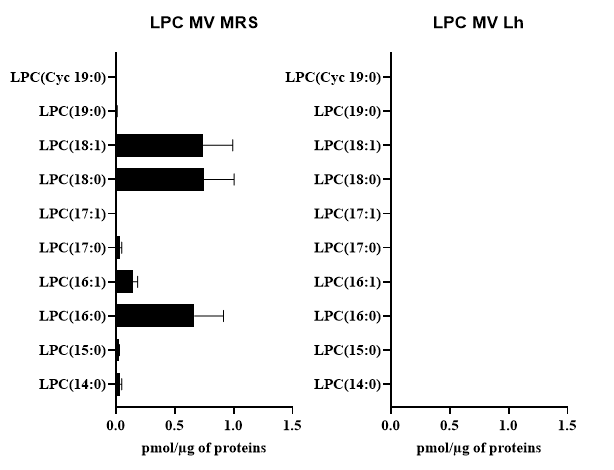
***

***Supplementary Figure 12. Lysophosphatidylcholine (LPC) species in L. helveticus VEL12193 MVs.*** Results are expressed as pmol per µg of protein. LPC were present only in MRS media MVs. Notably, LPC (Cyc 19:0) was not detected in both *L.helveticus* and MRS media MVs.


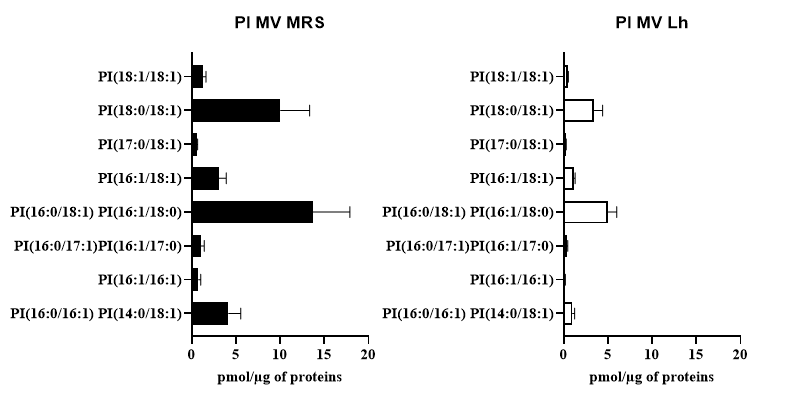


***Supplementary Figure 13. Phosphatidylinositols (PI) species in L. helveticus VEL12193 MVs.*** Results are expressed as pmol per µg of protein. PI were present in both MRS media and *L.helveticus* MVs. Cyc 19:0 esterified PI species were not detected in both *L. helveticus* and MRS media MVs.

***
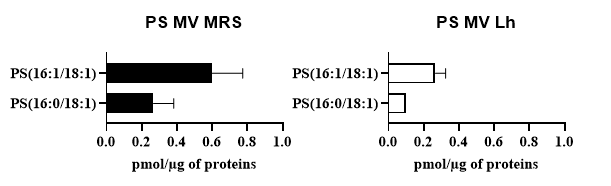
***

***Supplementary Figure 14. Phosphatidylserine (PS) species in L. helveticus VEL12193 MVs.*** Results are expressed as pmol per µg of protein. Only two forms of PS were detected in both MRS media and *L.helveticus* MVs. Cyc 19:0 esterified PS species were not detected in the MVs.


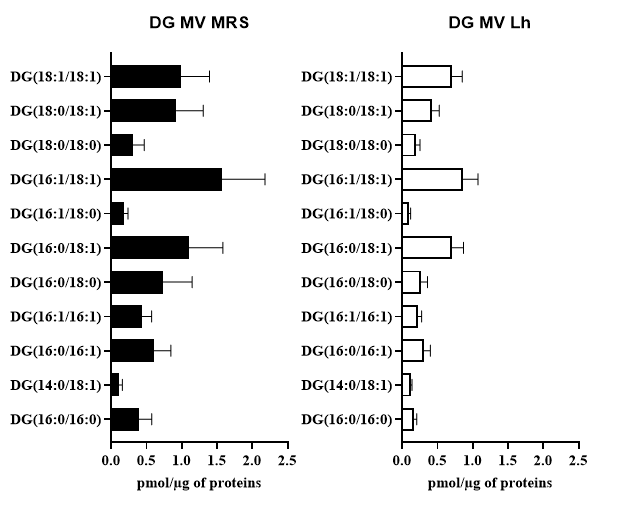


***Supplementary Figure 15. Diacylglycerols (DG) species in L. helveticus VEL12193 MVs.*** Results are expressed as pmol per µg of protein. DG were detected in both MRS media and *L.helveticus* MVs. Cyc 19:0 esterified DG species were not detected in the MVs.

***
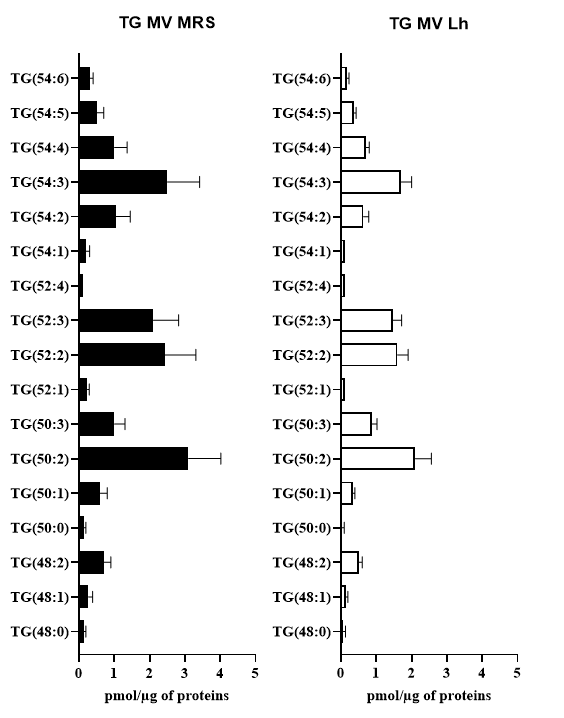
***

***Supplementary Figure 16. Triacylglycerols (TG) species in L. helveticus VEL12193 MVs.*** Results are expressed as pmol per µg of protein. TG were detected in both MRS media and *L.helveticus* MVs. Cyc 19:0 esterified TG species were not detected in the MVs.
